# Efficient production of guanosine in *Escherichia coli* by combinatorial metabolic engineering

**DOI:** 10.1186/s12934-024-02452-8

**Published:** 2024-06-19

**Authors:** Kun Zhang, Mengxing Qin, Yu Hou, Wenwen Zhang, Zhenyu Wang, Hailei Wang

**Affiliations:** Henan Province Engineering Laboratory for Bioconversion Technology of Functional Microbes, College of Life Sciences, Henan Normal University, Xinxiang, 453007 China

**Keywords:** Guanosine, *Escherichia coli*, Metabolic engineering, Integration expression, Metabolic flux

## Abstract

**Background:**

Guanosine is a purine nucleoside that is widely used as a raw material for food additives and pharmaceutical products. Microbial fermentation is the main production method of guanosine. However, the guanosine-producing strains possess multiple metabolic pathway interactions and complex regulatory mechanisms. The lack of strains with efficiently producing-guanosine greatly limited industrial application.

**Results:**

We attempted to efficiently produce guanosine in *Escherichia coli* using systematic metabolic engineering. First, we overexpressed the purine synthesis pathway from *Bacillus subtilis* and the *prs* gene, and deleted three genes involved in guanosine catabolism to increase guanosine accumulation. Subsequently, we attenuated *purA* expression and eliminated feedback and transcription dual inhibition. Then, we modified the metabolic flux of the glycolysis and Entner-Doudoroff (ED) pathways and performed redox cofactors rebalancing. Finally, transporter engineering and enhancing the guanosine synthesis pathway further increased the guanosine titre to 134.9 mg/L. After 72 h of the fed-batch fermentation in shake-flask, the guanosine titre achieved 289.8 mg/L.

**Conclusions:**

Our results reveal that the guanosine synthesis pathway was successfully optimized by combinatorial metabolic engineering, which could be applicable to the efficient synthesis of other nucleoside products.

**Supplementary Information:**

The online version contains supplementary material available at 10.1186/s12934-024-02452-8.

## Introduction

Guanosine and its nucleotide derivatives play essential physiological roles in nucleic acid synthesis, and possess antioxidant activity, neurotrophic and neuroprotective effects [1–3]. Additionally, guanosine has extensive applications as a crucial precursor for some medications used in the treatment of viral infections and tumors as well as food additives [4–6]. Guanosine production is mainly based on chemical synthesis, RNA enzymatic hydrolysis and microbial fermentation. Nevertheless, the first two approaches have the drawbacks of producing a large number of byproducts, complex separation and purification procedures, and high cost. Currently, microbial fermentation is the most commonly used approach for guanosine production. With the increasing demand for guanosine, a cost-effective method for the production of guanosine is needed. However, the lack of strains with high guanosine synthesis leads to low efficiency of guanosine production and high costs, hindering industrial application.

**Fig. 1:**
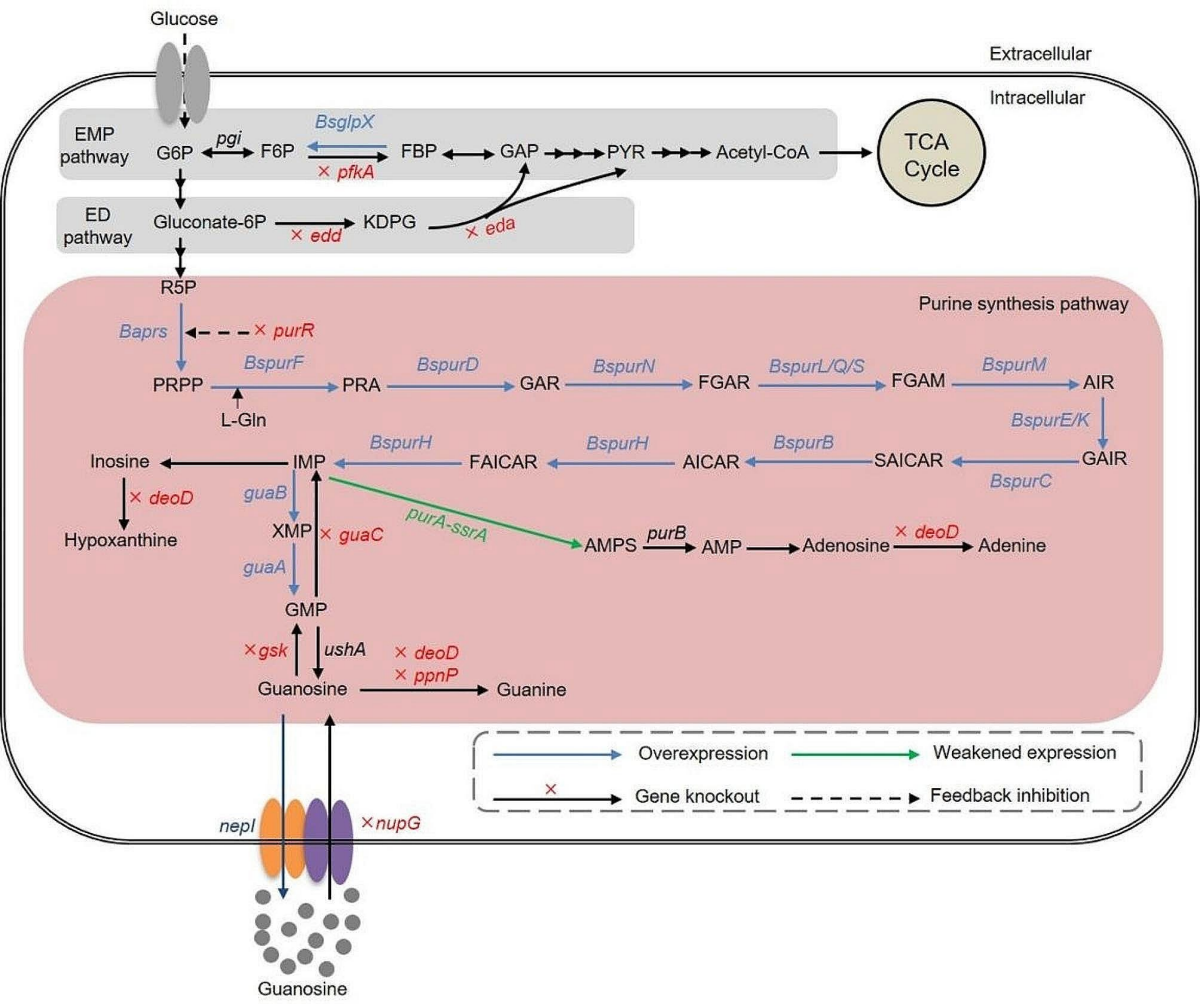
An overview of engineering strategies to increase guanosine production in *E. coli.* Blue and green arrows indicate overexpression and attenuation of the target genes. The red X indicates deletion of the corresponding gene. Dashed arrows indicate repression by regulatory protein. *Bs* indicates *Bacillus subtilis*. *Ba* indicates *Bacillus amyloliquefaciens*. Abbreviations: *pfkA*, 6-phosphofructokinase I; *BsglpX*, fructose-1,6-bisphosphatase II; *edd*, phosphogluconate dehydratase; *eda*, KHG/KDPG aldolase; *Baprs*, ribose-phosphate diphosphokinase; *BspurF*, glutamine PRPP amidotransferase; *BspurD*, phosphoribosylglycinamide synthetase; *BspurN*, THFA-dependent phosphoribosylglycinamide transformylases; *BspurQLS*, phosphoribosylformyl-glycinamidine synthetases I, II, and III; *BspurM*, phosphoribosylaminoimidazole synthetase; *BspurEK*, phosphoribosylaminoimidazole carboxylases I and II; *BspurC*, phosphoribosylaminoimidazolesuccinocarboxamide synthetase; *BsPurB*, adenylosuccinate lyase; *BspurH*, phosphoribosylaminoimidazole carboxamide formyltransferase and IMP cyclohydrolase; *purR*, DNA-binding transcriptional repressor; *purA*, adenylosuccinate synthase; *guaA*, GMP synthase; *guaB*, IMP dehydrogenase; *guaC*, GMP reductase; *deoD*, purine nucleoside phosphorylase; *ppnP*, nucleoside phosphorylase; *gsk*, inosine/guanosine kinase; *nupG*, nucleoside: H^+^ symporter; *nepI*, purine ribonucleoside exporter

Guanosine is synthesized though the Embden–Meyerhof–Parnas (EMP, or glycolysis) pathway, ED pathway, pentose phosphate pathway (PPP), and purine synthesis pathway (Fig. 1). First, the guanosine biosynthetic pathway in vivo begins with the formation of IMP and GMP. Subsequently, GMP is further converted to guanosine by phosphatase or 5’-nucleotidase. The *de novo* synthesis of guanosine includes 13 steps from 5′-phosphoribosyl pyrophosphate (PRPP), and precursors include bicarbonate, glycine, aspartate, glutamine, ribose-5′-phosphate (R5P), and some cofactors. In the salvage pathway, phosphoribosyltransferases can catalyze nucleobases to generate nucleotides. In *B. subtilis*, a gene cluster, *purEKB*-*purC*(orf)*QLF*-*purMNH*(*J*)-*purD*, constitutes the purine operon (*Bspur*) that contains three overlapping coding units and a single gene [7, 8]. Additionally, *purA*, *guaA*, and *guaB* are scattered across the genome as single genes. The dual regulation of the *Bspur* operon is mediated by transcription initiation and termination [9]. The *pur* operon exists as single genes or small operons across the chromosome in *E. coli*, and its expression is affected by the PurR repressor and the addition of guanine or hypoxanthine [10].

Native guanosine synthesis in bacteria is relatively complex and regulated by the EMP, ED and PPP, several branch and degradation pathways, feedback inhibition, etc. Guanosine anabolism requires a large quantity of purine precursors and considerable energy, and accumulating high levels of guanosine is difficult. Traditionally, chemical or physical mutagenesis methods have been used to generate strains with high guanosine yields. However, it is increasingly difficult to further increase the titre of guanosine based on existing guanosine-producing strains. Microbial cell factories constructed by system metabolic engineering provide an option for increasing the guanosine production. Moreover, such cell factories have been used to boost the production of uridine, inosine and guanosine [11–13]. Multiple engineering strategies including increasing the supply of precursor, disrupting the branch and degradation pathways, deleting the repressor PurR, overexpressing nucleoside efflux transporter PbuE, constructing an unnecessary protein-reduced chassis, and building genome-scale metabolic network models, have been applied to promote the synthesis of purine nucleosides [11, 14–16]. *E. coli* was applied to synthesize up to 7.5 g/L inosine by overexpression of the desensitized *prs* and *purF* genes and knockout of the *purA*, *purR*, *deoD*, and *purF* genes [17, 18]. Deletion of the *gsk* gene encoding inosine/guanosine kinase led to increased guanosine accumulation [19]. It’s reported that the guanosine production in engineered *E*. *coli* and *B*. *subtilis* was 120 mg/L and 115.2 mg/L in shake flask, respectively [13, 19]. Overall, previous studies focused on several genetic modifications of synthesis pathways of purine nucleoside. Employing a systems metabolic engineering strategy to construct engineered strains is necessary for the effective production of guanosine.

As a model microorganism, *E. coli* is a promising chassis strain for guanosine synthesis. Multiple gene editing tools and synthetic biology and metabolic engineering strategies are favourable for constructing cell factories [20]. CRISPR/Cas9-mediated homologous recombination is a precise gene editing method that can achieve stable targeted gene knockout or integration [21]. To enhance the guanosine titre, we adopted a systematic metabolic engineering strategy to modify the chassis strain *E. coli* MG1655. First, overexpression of the purine operon *Bspur* and the *prs* gene was carried out to enhance the flux into guanosine synthesis. Then, the guanosine degradation pathway was blocked to reduce guanosine consumption. Subsequently, branch pathway for adenosine synthesis, feedback inhibition from repressor PurR, EMP and ED pathways were modified to drive more carbon flux into guanosine synthesis. Next, redox cofactors rebalancing, transporter engineering and enhancing the guanosine synthesis pathway were used to promote guanosine accumulation. Finally, the best strain MQ39 produced 289.8 mg/L guanosine in fed-batch fermentation in shake flasks after 72 h. The constructed cell factory of guanosine production in this study can also be served as chassis strains to synthesize other valuable purine nucleosides like adenosine and inosine.

## Materials and methods

### Strains, plasmids, and cultivation conditions

*E. coli* DH5α was used for vector construction, and *E. coli* MG1655 Δ*lacI* was used as the starting chassis strain to perform genomic editing. Plasmids #1 and #2 for the CRISPR/Cas9 genome editing were kindly supplied by Prof. Huo and Dr. Huang of Beijing Institute of Technology [21]. Plasmid #1 consists of a *p15A* replication origin, a kanamycin resistance gene *Kan*^*R*^, the sucrose-sensitive *sacB* gene, a *CAS9* expression system induced by L-arabinose and an isopropyl-β-D-thiogalactopyranoside (IPTG) inducible λ-Red (Gam, Beta, and Exo) recombination system. Plasmid #2 includes a temperature-sensitive *pSC101* replication origin, the promoter P_*BAD*_-N20-gRNA scaffold expressing cassette for Cas9 binding, a donor DNA-generation system serving as an editing template and the ampicillin resistance gene *Amp*^*R*^. The *sacB* gene of plasmid #1 and the *pSC101* replication origin of plasmid #2 were used for plasmid curing. The strains and plasmids used in this study are listed in Table 1. *E. coli* DH5α and MG1655 Δ*lacI* were cultured in Luria–Bertani (LB) medium at 37 °C. Ampicillin (100 µg/mL) and kanamycin (50 µg/mL) were added to the cultures when needed. IPTG, L-arabinose, glucose, and sucrose were added at concentrations of 1 mM, 20 mM, 20 g/L and 20 g/L, respectively.

**Table 1:** Strains and plasmids used in this study

Strains and plasmids	Description	Sources
Strains		
*E. coli* DH5α	the cloning host	This lab
*E. coli* MG1655 Δ*lacI*	the starting strain	This lab
MQ1	*E. coli* MG1655, Δ*lacI*, *yghX*::P_*trc*_-*BspurEK*-T_*BBa_B1006*_	This study
MQ2	MQ1, *ypjC*-*ileY*::P_*trc*_-*BspurBCSQ*-T_*BBa_B1002*_	This study
MQ3	MQ2, *fliK*::P_*trc*_-*BspurLF-*T_*BBa_B1004*_	This study
MQ4	MQ3, *lacZ*::P_*trc*_-*BspurMNHD*-T_*rrnB T2*_	This study
MQ5	MQ4, *aslA*-*glmZ*::P_*trc*_-*prs* -T_*BBa_B1005*_	This study
MQ6	MQ4, *aslA*-*glmZ*::P_*trc*_-*Ecprs*^*D128A*^-T_*BBa_B1005*_	This study
MQ7	MQ4, *aslA*-*glmZ*::P_*trc*_-*Baprs*-T_*BBa_B1005*_	This study
MQ9	MQ7, Δ*deoD*	This study
MQ14	MQ9, Δ*ppnP*	This study
MQ15	MQ9, Δ*gsk*	This study
MQ16	MQ9, Δ*rihA*	This study
MQ17	MQ9, Δ*rihB*	This study
MQ18	MQ9, Δ*rihC*	This study
MQ19	MQ14, Δ*gsk*	This study
MQ20	MQ19, Δ*purA*	This study
MQ21	MQ19, Δ*purA*::P_*trc*_-*BspurA*^*P242N*^-T_*BBa_B1003*_	This study
MQ23	MQ19, *purA*:: *purA*-*ssrA*	This study
MQ24	MQ23, Δ*purR*	This study
MQ25	MQ24, Δ*pfkA*	This study
MQ26	MQ25, Δ*pfkA*::P_*J23116*_-*glpX*-T_*BBa_B1003*_	This study
MQ27	MQ25, Δ*pfkA*::P_*J23116*_-*BsglpX*-T_*BBa_B1003*_	This study
MQ28	MQ27, Δ*eda*, Δ*edd*	This study
MQ29	MQ28, Δ*pntAB*	This study
MQ30	MQ28, Δ*yghE*::P_*trc*_-*sthA*-T_*BBa_B1010*_	This study
MQ31	MQ28, Δ*pntAB*::P_*trc*_-*sthA*-T_*BBa_B1010*_	This study
MQ32	MQ28, Δ*nupG*	This study
MQ33	MQ28, Δ*nupG*::P_*J23108*_*-nepI-*T_*BBa_B1003*_	This study
MQ34	MQ28, Δ*nupG*::P_*J23108*_*-BspbuE-*T_*BBa_B1003*_	This study
MQ38	MQ33, *ykgH*-*betA*::P_*tac*_-*guaAB*	This study
MQ39	MQ33, Δ*guaC*::P_*tac*_-*guaAB*	This study
Plasmids		
#1	*Kan*^*R*^, Cas9 and λ-Red recombinase expression vector	[21]
#2	*Amp*^*R*^, gRNA expression vector	[21]

### Construction of plasmids and recombinant strains

gRNA expression plasmids were constructed based on plasmid #2 and used for gene deletion and genomic integration. The purified two homologous arms (~ 500 bp) and the inserted expression cassette were fused by overlap extension PCR to obtain donor DNA. Subsequently, the two fragments from the plasmid #2 backbone and donor DNA were ligated with the ClonExpress II One Step Cloning Kit (Vazyme, Nanjing, China) to form the specific gRNA plasmid #2.

The recombinant strains were obtained according to a previous protocol [21]. Briefly, the specific gRNA plasmid #2 was introduced into the MG1655Δ*lacI* strain harbouring plasmid #1for gene knockout and chromosomal integration. Colonies grown on the plates were verified by colony PCR, and the correct colonies were confirmed by DNA sequencing. Both plasmids #1 and #2 could be cured by cultivation on LB solid plate containing 2% sucrose at 37 °C for 24 h when the obtained strain was not required to further genetic modifications. The primers are listed in Supplementary Table S1. The integration expression cassettes and a flowchart of gene editing for generating the engineered strains are shown in Supplementary Figs. S1 and S2.

## Fed-batch fermentation

For guanosine fermentation, fresh single colonies of engineered strains were inoculated in 10 mL of liquid LBG medium overnight at 37 °C and 220 rpm. Then, the seed cultures of 3 mL were transferred into 27 mL of LBG (LB plus 2% glucose) medium in a 500-mL shake flask and cultured for 72 h at 37 °C and 220 rpm. Fed-batch fermentation was almost the same as the batch method. During the fed-batch process, the pH was kept at approximately 7.0 by adding ammonia with a microinjector, and phenol red was used as the pH indicator. Glucose solution (30%) was added for fed-batch culture when the glucose is depleted.

### Substrate and product analysis

Optical density of the engineered strains was measured by the ultraviolet spectrophotometer at 600 nm. The glucose concentration was analysed by the 3,5-dinitrosalicylic acid (DNS) method. Cells were collected and centrifuged at 12,000 rpm for 10 min. The resultant supernatants were filtered through 0.22 μm syringe filters for the next analysis. The guanosine and hypoxanthine concentrations were quantified by HPLC (Agilent 1200) using a RD-C18 5 μm column (Zhongpu scientific, China) using the mobile phase methanol/water mixture (15:85 v/v) with a flow rate of 1 mL/min. The detection temperature and wavelength were set to 25 °C and 254 nm, respectively.

### Statistical analysis

All experiments were performed with four independent cultures. Statistical significance was analysed by a two-tailed Student’s t-test. p value < 0.05 (*) was defined as statistical difference; p value < 0.01 (**) was defined as significant; p value < 0.001 (***) was defined as highly significant; ns was defined as no significant difference.

## Results

### Increasing the metabolic flux and precursor PRPP supply

**Fig. 2:**
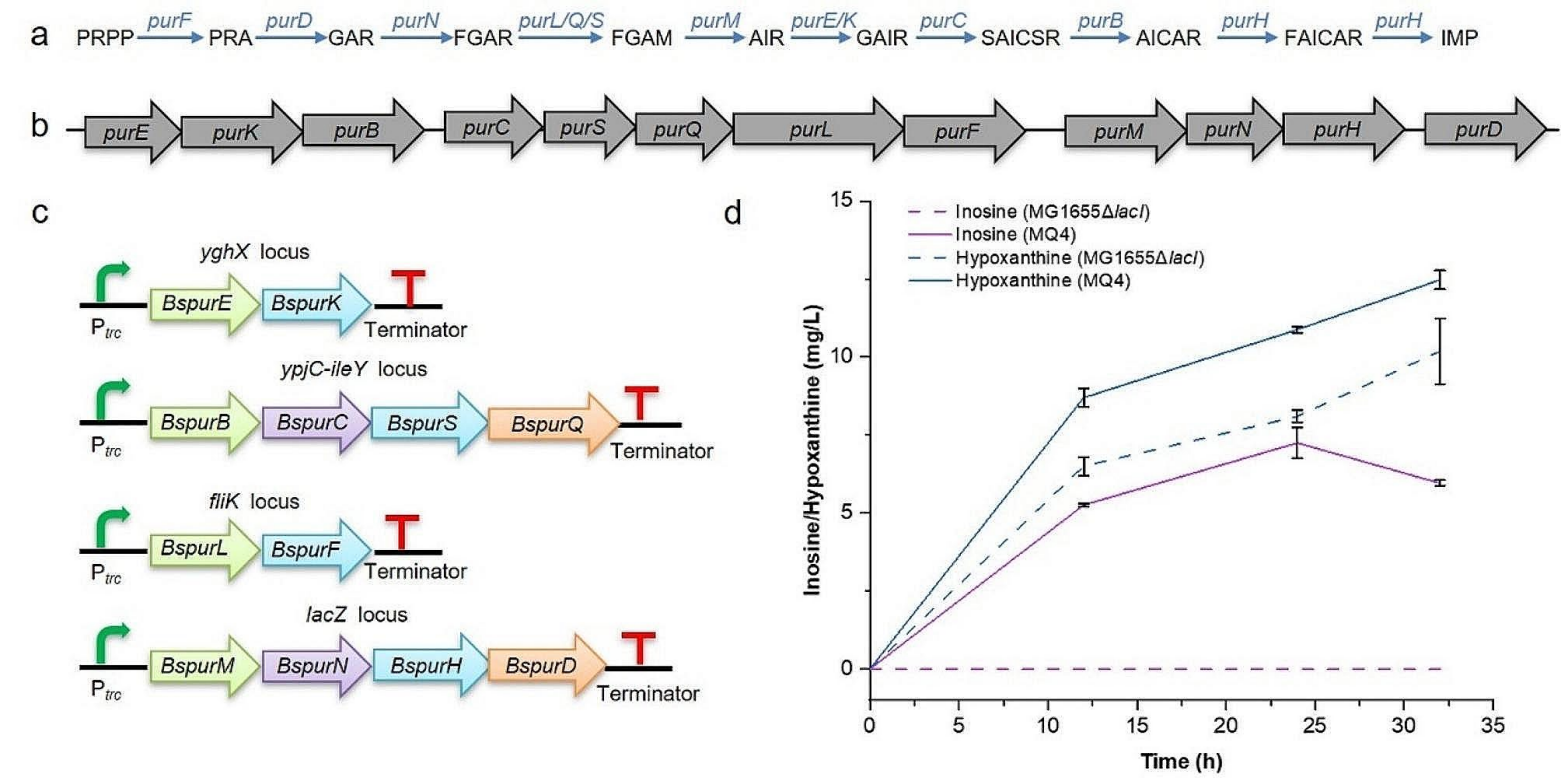
Overexpressing the purine synthesis operon from *B*. *subtilis*. (a) The native metabolic pathway of purine biosynthesis in *E*. *coli*; (b) Structural gene diagram of the purine operon in *B. subtilis*; (c) Integrated expression cassettes of the purine operon; (d) Production of hypoxanthine and inosine by overexpressing the purine synthesis operon. All data represent the mean ± s.d. (*n* = 4 biologically independent samples). Error bars were analysed by Student’s t test (two-sample, two-tailed; ** p value < 0.01, *** p value < 0.001)

To increase the metabolic flux of guanosine synthesis, we sought to introduce the purine synthesis pathway of *B. subtilis* 168 into chromosome of *E. coli*. However, the complete purine synthesis pathway *Bspur* operon contains 11 genes (~ 12 kb) without *purR* (Fig. 2a and b). It is quite difficult for chromosomal integration expression for large DNA fragments by the CRISPR/Cas9-mediated homologous recombination. With increasing length of the integrated fragment, the recombination efficiency significantly decreases, which is not conducive to obtaining positive clones. Therefore, the *Bspur* operon was divided into four parts, each 1000–5000 bp. These parts were controlled by the P_*trc*_ promoter and different synthetic terminators to generate expression cassettes (Fig. 2b). Four expression cassettes were further flanked by upstream and downstream homologous arms to form the donor DNA. Meanwhile, we deleted the *lacI* gene to eliminate regulation of the LacI repressor on the P_*trc*_ promoter, and the resultant strain MG1655 Δ*lacI* was used as the starting chassis. After integration of the donor DNA into different loci located on the chromosome, the engineered *E. coli* strain MQ4 overexpressing the *Bspur* operon was obtained. The results showed that MG1655 Δ*lacI* strain did not produce inosine, while the MQ4 strain achieved the highest inosine production at 7.3 mg/L after 24 h of fermentation, indicating that *Bspur* operon integration is beneficial for increasing the metabolic flux of purine synthesis in *E*. *coli* (Fig. 2d). In addition, MG1655 Δ*lacI* and MQ4 strains did not produce guanosine (data not shown). MG1655 Δ*lacI* and MQ4 accumulated 8.1 mg/L and 10.9 mg/L of hypoxanthine after 24 h of fermentation, respectively, and the amount of hypoxanthine still showed an increasing trend. The final OD_600_ of MQ4 was very similar to that of MG1655 Δ*lacI.*

**Fig. 3:**
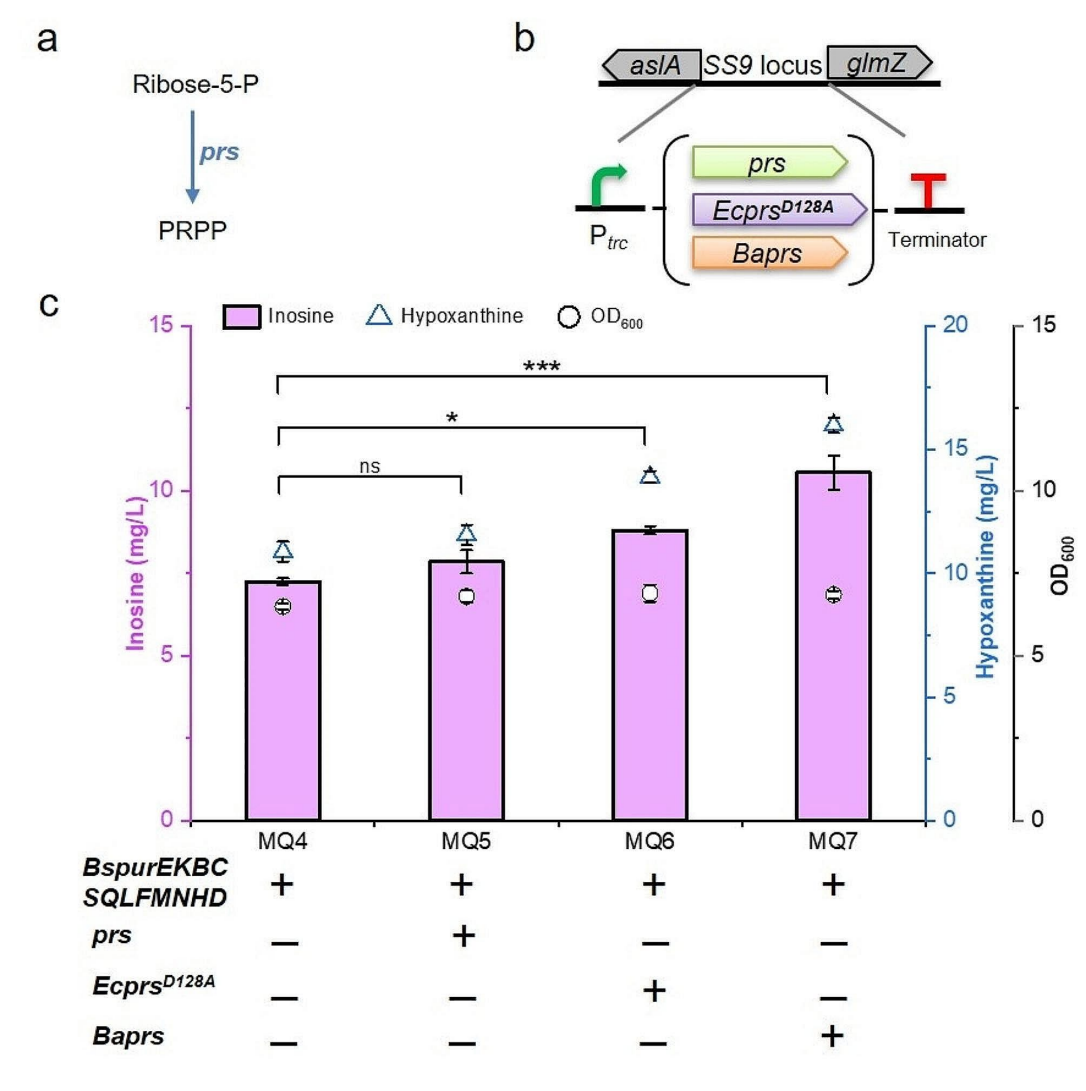
Overexpressing the *prs* gene for guanosine production. (a) Schematic of the PRPP synthesis pathway in *E*. *coli*; (b) Integrated expression cassettes of the *prs* gene; (c) Production of hypoxanthine and inosine by overexpressing the *prs* gene. All data represent the mean ± s.d. (*n* = 4 biologically independent samples). Error bars were analysed by Student’s t test (two-sample, two-tailed; ** p value < 0.01, *** p value < 0.001)

PRPP is a crucial precursor for purine and pyrimidine nucleoside synthesis in vivo. PRPP is generated from R5P and ATP, which is catalysed by PRPP synthase (Figs. 1 and 3a). PRPP synthase is regulated by feedback inhibition via ADP, and mutated PRPP synthase can relieve nucleotide inhibition [17]. The *prs* gene from *E. coli*, the mutated *Ecprs*^*D128A*^ gene and the *Baprs* gene from *Bacillus amyloliquefaciens* were integrated into the *aslA*-*glmZ* locus of MQ4 (Fig. 3b), respectively. Guanosine and adenosine were not detected by HPLC in MQ5, MQ6, and MQ7. The production of inosine in MQ5, MQ6, and MQ7 was 7.8 mg/L, 8.8 mg/L, and 10.6 mg/L, respectively, which increased by 6.8%, 20.5%, and 45.2% compared to the MQ4 strain. In addition, MQ5, MQ6 and MQ7 strains accumulated 11.6 mg/L, 13.9 mg/L, and 16.0 mg/L of hypoxanthine, respectively (Fig. 3c). These results implied that although the flux of inosine synthesis was further enhanced by the overexpression of the *prs* gene, the guanosine concentration was possibly too low to be monitored by HPLC. In the *de novo* synthesis pathway of purine nucleosides, IMP requires a one-step reaction to generate inosine, while the conversion of IMP to guanosine and adenosine requires a three-step reaction. We speculate that IMP may be used to preferentially synthesize inosine, and the metabolic flux of the inosine synthesis pathway is higher than that of guanosine. In addition, guanosine is possibly degraded in *E. coli.*

## Blocking the degradation pathway

**Fig. 4:**
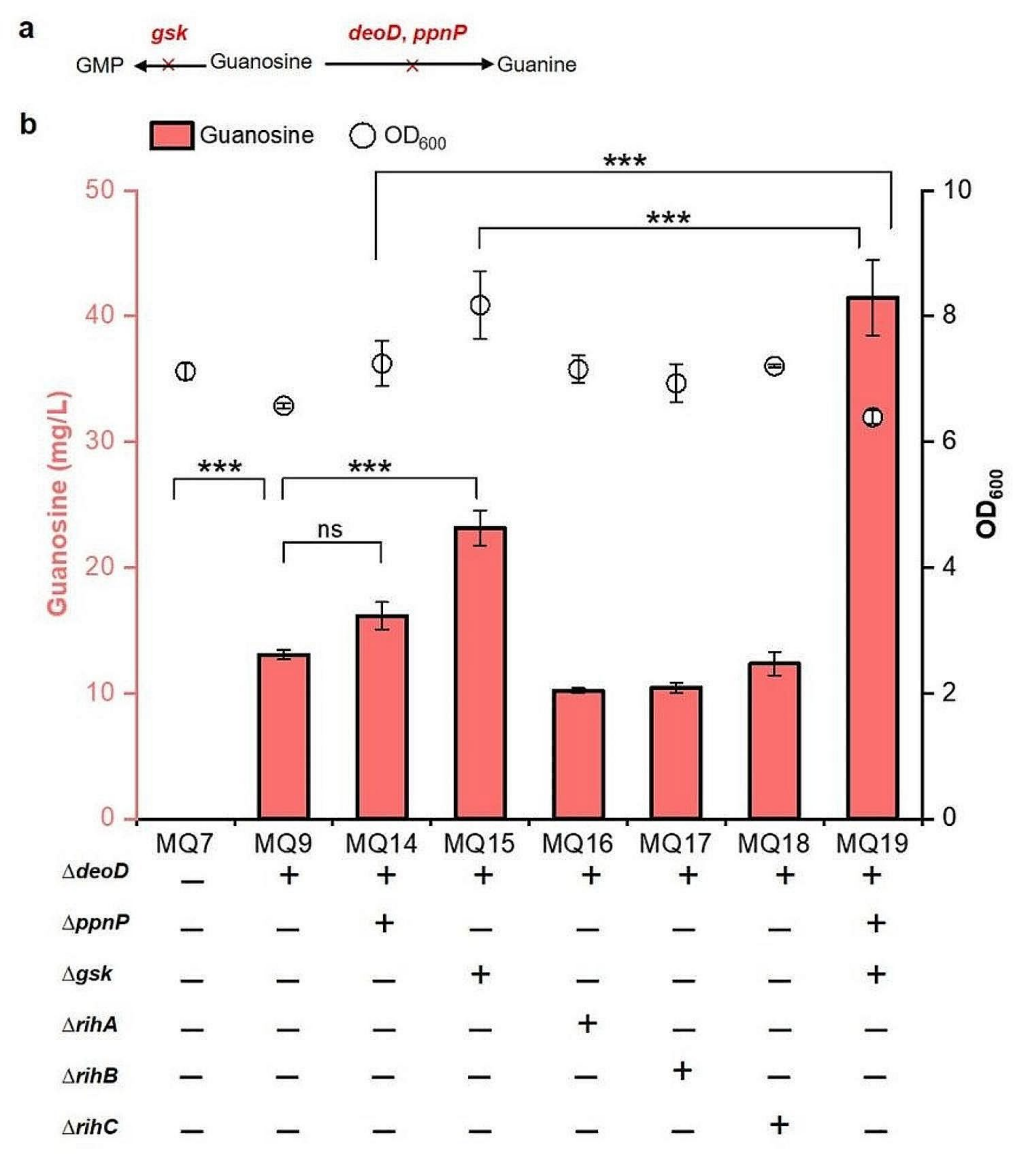
Disruption of guanosine catabolism-related genes. (a) The related pathways of guanosine catabolism in *E. coli*. (b) Production of guanosine by deleting guanosine catabolism-related genes. All data represent the mean ± s.d. (*n* = 4 biologically independent samples). Error bars were analysed by Student’s t test (two-sample, two-tailed; *** p value < 0.001)

Guanosine can be degraded by multiple cellular enzymes, including inosine/guanosine kinase encoded by *gsk* gene, two guanosine phosphorylases encoded by *deoD* and *ppnP* genes, and three nucleoside hydrolases encoded by *rihA*, *rihB*, and *rihC* genes [19, 22] (Fig. 4a). Notably, phosphorylase DeoD can also degrade inosine and adenosine into hypoxanthine and adenine, respectively. These enzymes were selected to be blocked in *E. coli*. The results showed that MQ9 (*deoD* deletion) produced 13.1 mg/L guanosine after 72 h of fermentation (Fig. 4b). After further knockout of *ppnP* and *gsk*, the titre of guanosine in MQ14 and MQ15 reached 16.2 and 23.2 mg/L, respectively. However, the production of guanosine in the MQ16 (*rihA* deletion), MQ17 (*rihB* deletion) and MQ18 (*rihC* deletion) strains was obviously decreased, indicating that the deletion of *rihA*, *rihB* and *rihC* is unfavourable for guanosine accumulation. Subsequently, MQ19 (triple deletion of *deoD*, *ppnP* and *gsk*) accumulated 41.5 mg/L guanosine after 72 h fermentation, an increase of 78.9% compared with MQ15 (Fig. 4b). These results suggested that disrupting the degradation pathway is beneficial for the accumulation of guanosine in *E. coli*.

### Down regulating *purA* expression and removing feedback and transcription dual inhibition

**Fig. 5:**
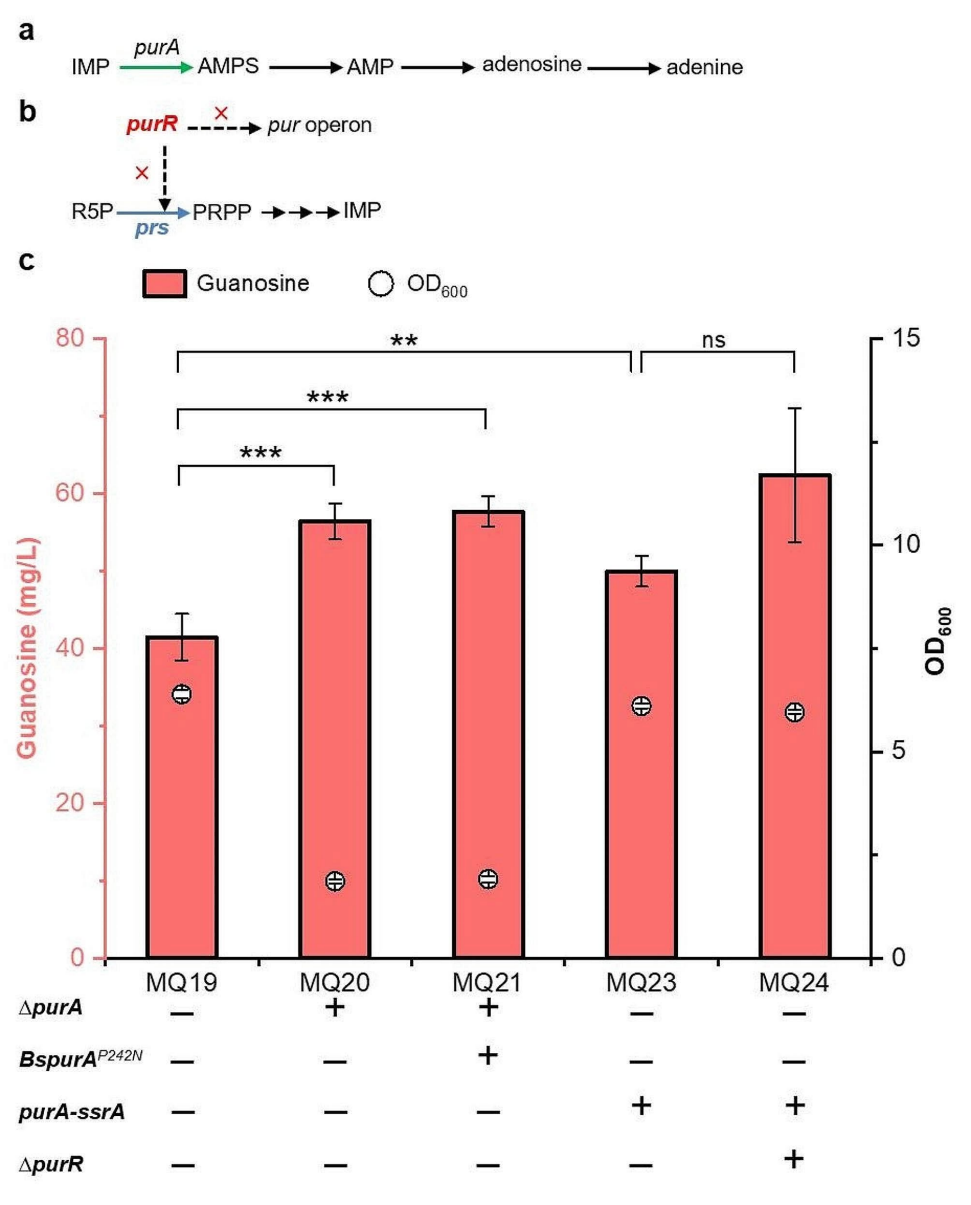
Adjusting the metabolic flux from adenosine synthesis and the feedback inhibition of PurR. The intrinsic pathway of adenosine synthesis (a) and the feedback inhibition mediated by PurR (b); (c) Production of guanosine in the engineered strains. All data represent the mean ± s.d. (*n* = 4 biologically independent samples). Error bars were analysed by Student’s t test (two-sample, two-tailed; ** p value < 0.01, *** p value < 0.001, ns represents no significant difference)

The conversion of IMP to adenosine was catalysed by adenylosuccinate synthetase (ADSS), which is a competitive pathway for guanosine production (Fig. 5a). Given that adenosine is important for cell growth, the adenosine synthesis pathway was attenuated to direct more carbon flux towards guanosine synthesis. The *ssrA* degradation tag (DAS + 4) was added to the C-terminus of ADSS encoded by *purA* gene to induce cytoplasmic degradation of ADSS [23]. Additionally, the native *purA* was replaced with *BspurA*^*P242N*^ to lower the flux towards adenosine branch from IMP. The guanosine titres of MQ20 (*purA* disruption), MQ21 (*BspurA*^*P242N*^) and MQ23 (*purA-ssrA*) were 56.3, 57.7 and 50.0 mg/L, respectively (Fig. 5c). However, the final OD_600_ of the MQ20 and MQ21 strains was 1.9, which was significantly reduced by 70.3% compared with the MQ19 strain. The biomass of MQ23 strain is similar to that of MQ19 strain, and the guanosine production increased by 20.4%. Considering that OD_600_ is an important factor for strain engineering and fermentation, the MQ23 strain was selected for subsequent genetic modification. These results suggested that the *purA* gene is indispensable for cell growth and that its deletion can lead to severe growth deficiency.

The PurR repressor can inhibit the transcription of the *pur* operon and the *prs* gene (Fig. 5b). We sought to delete the *purR* gene to remove the repression regulation and enhance the intracellular concentration of PRPP. The results showed that MQ24 (*purR* deletion) accumulated 62.4 mg/L guanosine, an increase of 24.8% compared with MQ23 (Fig. 5c).

### Redistributing the metabolic flux of the EMP and ED pathways and balancing redox cofactors

**Fig. 6:**
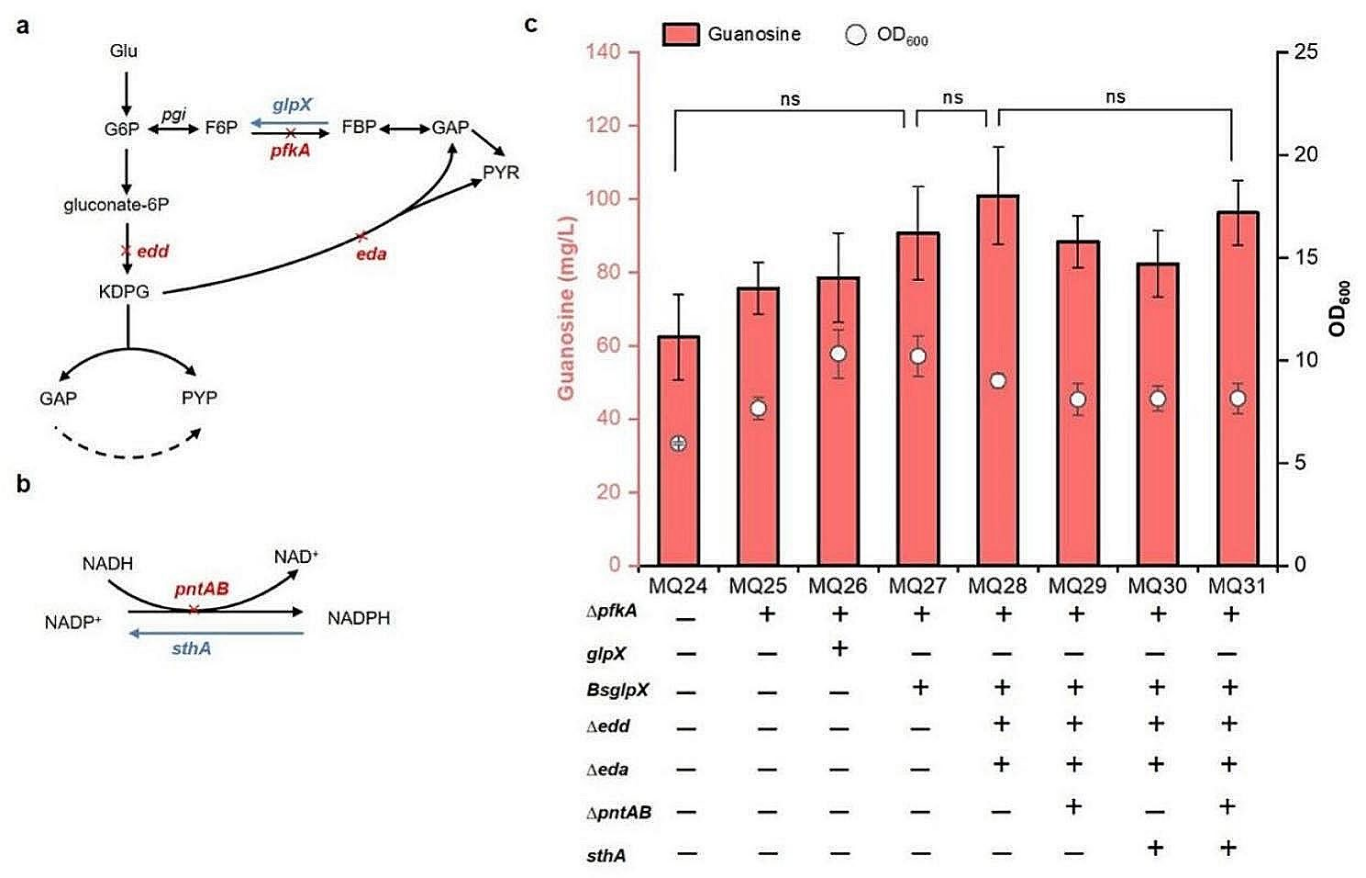
Redistributing the metabolic flux of EMP and ED and redox cofactor rebalancing for the accumulation of guanosine. (a) Schematic of the EMP and ED pathways; (b) Schematic of redox cofactor rebalancing; (c) Increased guanosine production by downregulating the metabolic flux of EMP and ED and redox cofactor rebalancing. All data represent the mean ± s.d. (*n* = 4 biologically independent samples). Error bars were analysed by Student’s t test (two-sample, two-tailed; ns represents no significant difference)

Glucose-6-phosphate (G6P) is an important intermediate of guanosine production as well as a component of the EMP and ED pathways. The biosynthetic pathway of purine competes for metabolic flux with the glycolysis and ED pathways (Fig. 6a). Nevertheless, the disruption of glycolysis and the ED pathway may lead to metabolic imbalance and sever growth defect. The carbon flux between the EMP and purine synthesis pathways should be efficiently distributed to achieve higher guanosine production. In glycolysis, the Pfk enzyme catalyzes the conversion of fructose-6-phosphate (F6P) to fructose-1,6-diphosphate (FBP). Pfk has two isoenzyme forms, and 6-phosphofructokinase I (Pfk-I, encoded by *pfkA*) possesses more than 90% of Pfk enzymatic activity. Fbpase, including Fbpase I and Fbpase II, can hydrolyse FBP to F6P (Fig. 6a). Compared with Fbpase I (encoded by *fbp*), Fbpase II, encoded by *glpX*, exhibits reduced sensitivity towards feedback inhibition of G6P [24, 25]. In the ED pathway, phosphogluconate dehydratase encoded by *edd* and 2-keto-3-deoxygluconate-6-phosphate aldolase encoded by *eda* convert 6-phosphogluconate (6-PG) into pyruvate and glyceraldehyde-3-phosphate.

To enhance flux into the PPP towards the production of guanosine, we deleted the *pfkA* gene and overexpressed *glpX* and *BsglpX* using the weak synthetic promoter P_*J23116*_ to adjust the carbon flux of the glycolysis and ED pathways. The guanosine titres of MQ25 (*pfkA* deletion), MQ26 (*pfkA* deletion plus *glpX* overexpression) and MQ27 (*pfkA* deletion plus *BsglpX* overexpression) reached 75.6, 78.6 and 90.7 mg/L, respectively (Fig. 6c). Compared to the MQ24 strain, the production of guanosine was increased by 21.2%, 26.0% and 45.4%, respectively. The ED pathway was also interrupted by double knockout of the *edd* and *eda* genes. The resultant MQ28 strain accumulated 100.9 mg/L guanosine, an increase of 11.2% compared with MQ27 (Fig. 6c). Engineering the EMP and ED pathways displayed a synergistic effect for guanosine accumulation. These results proved that attenuating the flux of the EMP and ED pathways is a useful strategy for enhancing guanosine synthesis.

Although adjusting glycolysis and the ED pathway can improve guanosine production, it might result in cofactor imbalance. The inactivation of *pfkA*, *edd*, and *eda* and overexpression of *glpX* can redirect more carbon flux into the PPP, which can lead to excess NADPH and NADH deficiency [26]. To restore cofactor balance, soluble pyridine nucleotide transhydrogenase SthA (UdhA), encoded by *sthA*, was introduced into the genome to enhance the conversion of NADPH to NADH, and the membrane-bound transhydrogenase encoded by the *pntAB* gene was deleted to decrease the conversion of NADH to NADPH (Fig. 6b). The guanosine titres of MQ29 (*pntAB* deletion), MQ30 (*sthA* overexpression) and MQ31 (*pntAB* deletion and *sthA* overexpression) were 88.3, 82.3 and 96.3 mg/L, respectively (Fig. 6c). Redox cofactor rebalancing failed to further improve the production of guanosine, which suggested that the NADH and NADPH levels in MQ28 might be suitable for guanosine synthesis.

### Transporter engineering for guanosine accumulation

**Fig. 7:**
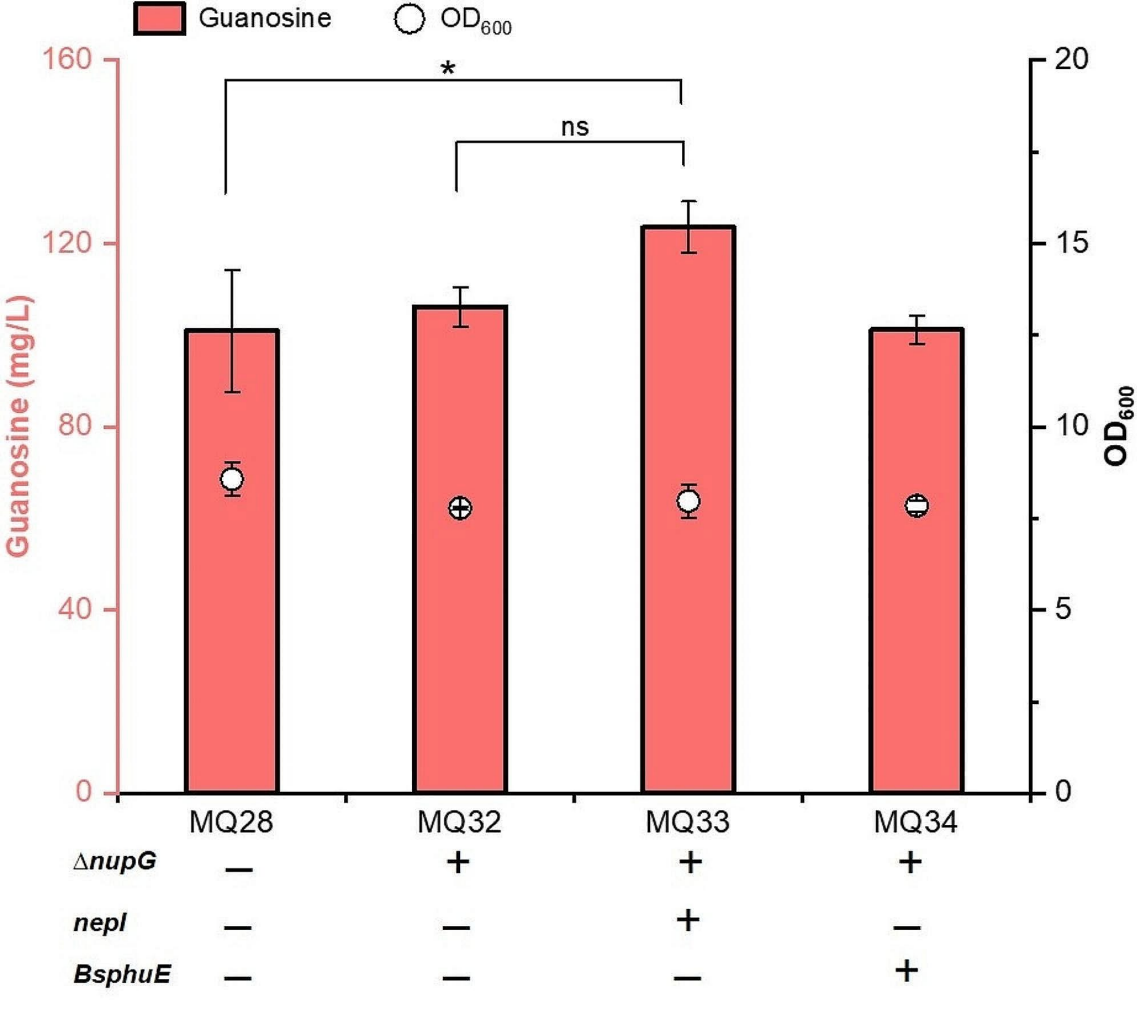
Engineering transporters for guanosine production. All data represent the mean ± s.d. (*n* = 4 biologically independent samples). Error bars were analysed by Student’s t test (two-sample, two-tailed; *p value < 0.05)

Excessive accumulation of nucleosides leads to metabolic burden on the cell and causes product-mediated feedback inhibition. Previous studies illustrated that the NupG protein of *E. coli* can transport extracellular nucleosides into cells, and knockout of the *nupG* and *nupC* genes can increase the uridine and cytidine titres [11, 27, 28]. Meanwhile, overexpression of the nucleoside efflux transporters PbuE and NepI notably increased inosine secretion [29]. Transporters are essential for the hyperproduction of target products. To investigate the effects of these transporters on guanosine synthesis, we deleted the *nupG* gene in MQ28 to obtain strain MQ32. The inactivation of the *nupG* gene led to a slight increase in the guanosine titre (Fig. 7). Subsequently, overexpression of *nepI* and *BspbuE* in MQ32 generated the MQ33 and MQ34 strains, respectively. The guanosine titre of MQ33 was 123.6 mg/L, which was 16.4% higher than that obtained from MQ32. Blockage and overexpression of nucleoside transporters can prevent the absorption of exogenous guanosine and facilitate the extracellular outflow of guanosine [30, 31]. The results demonstrated that engineering transporters can enhance the performance of chassis strains by facilitating efflux and decreasing the accumulation of the desired intracellular products.

### Strengthening the guanosine synthesis pathway

**Fig. 8:**
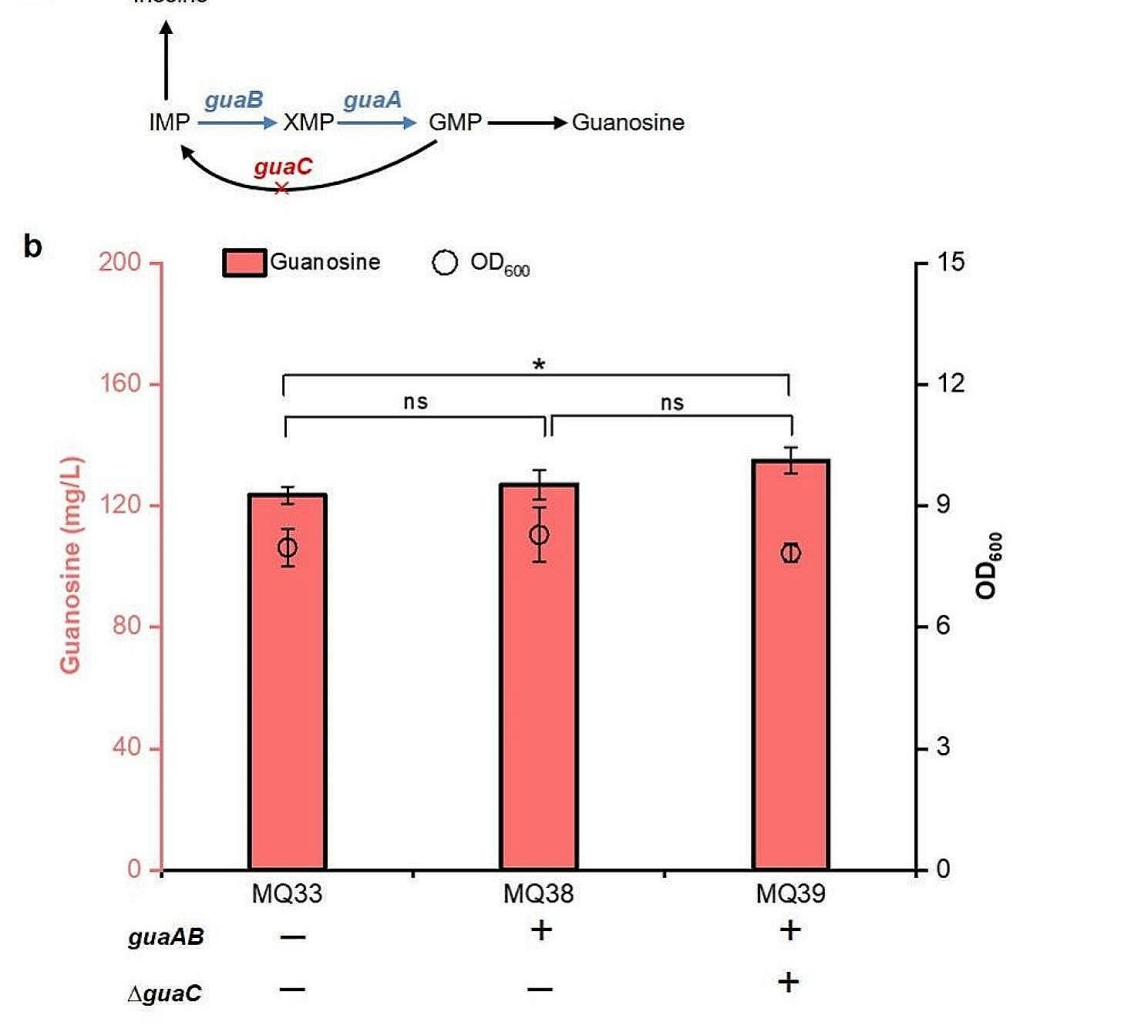
The effect of engineering the guanosine synthesis pathway. (a) Schematic of guanosine synthesis pathway; (b) Effects of strengthening the guanosine synthesis pathway on guanosine accumulation. All data represent the mean ± s.d. (*n* = 4 biologically independent samples). Error bars were analysed by Student’s t test (two-sample, two-tailed; *p value < 0.05)

The reduction reaction from GMP to IMP is catalyzed by GMP reductase encoded by *guaC* gene, and overexpression of *guaA* and *guaB* genes is beneficial to guanosine synthesis. To favor guanosine production, we integrated the *guaAB* gene into *ykgH*-*betA* locus and *guaC* locus to obtain strains MQ38 and MQ39. These two strains accumulated 127.0 mg/L and 134.9 mg/L guanosine, respectively (Fig. 8). These results illustrated that enhancing the synthesis pathway of guanosine is conducive to guanosine accumulation.

### Guanosine production in shake flasks

**Fig. 9:**
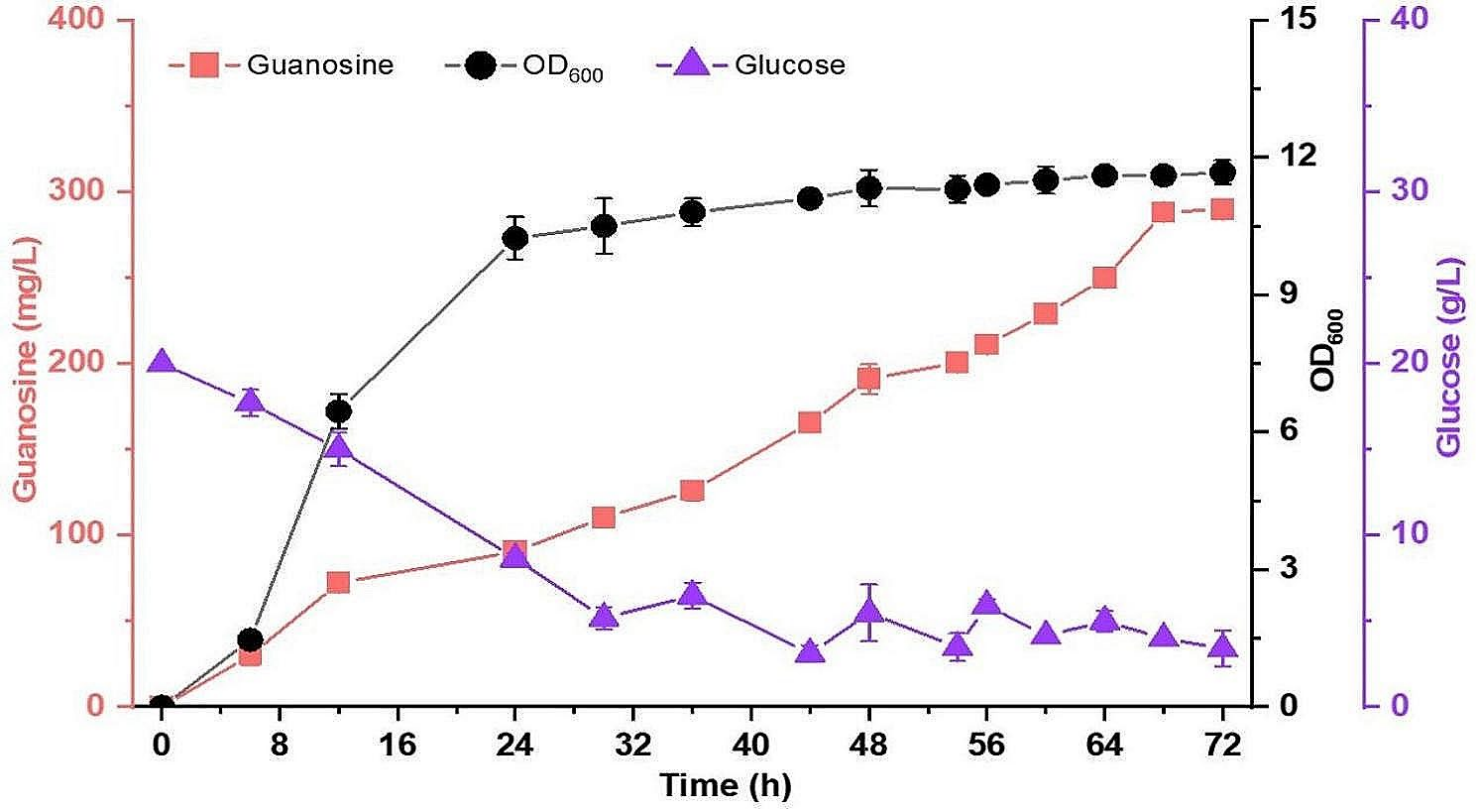
Assessment of guanosine production using the strain MQ39. Fed-batch fermentation was performed in shake flasks. Four biological replicates were performed, and the error bars indicate the standard deviation

The strain MQ39 was grown in shake flasks to evaluate the performance of guanosine production under fed-batch fermentation. The cells grew rapidly from the initiation of fermentation, and the OD_600_ reached 11.7 at 72 h (Fig. 9). The production of guanosine gradually increased in the whole fermentation process, representing a cell growth-independent production trend. The guanosine titre reached 289.8 mg/L with a yield of 9.89 mg/g glucose after fermentation for 72 h.

## Discussion

Guanosine and other purine nucleosides are crucial for various cellular physiological processes and have broad applications in the fields of antitumor/antiviral medication as well as food additives. Nevertheless, microbial production of guanosine is still a large challenge because of multiple metabolic pathway interactions and complex regulatory mechanisms. Here, we successfully constructed a microbial cell factory for efficient guanosine production by combinational metabolic engineering strategies. The engineered strains can produce 289.8 mg/L guanosine after 72 h of fermentation.

Low metabolic flux and insufficient precursor supply are the main limiting factors for synthesizing target products in microbial hosts [32]. Overexpression of homologous or heterologous metabolic pathways is an effective method to enhance the metabolic flux for target product production [33]. Previous methods have mainly focused on plasmid expression. The advantage of plasmid expression is that more copies of the target genes possibly lead to high expression levels. However, it is necessary to add amino acids or antibiotics to prevent plasmid loss, and the continuous addition of antibiotics may increase the cost of fermentation. In addition, a large recombinant plasmid may bring about a metabolic burden on the strain and result in growth delay, which might be unsuitable for constructing pyrimidine-producing strains [11]. Plasmid loss is also easily observed in fed-bath cultivation, which results in ineffective consumption of carbon sources and reduced product titre [26]. Integration of the desired metabolic pathway into the genome is a stable overexpression strategy for multiple genes [34]. Chromosomal integration of multiple genes possesses several advantages, such as achieving stable gene expression, avoiding vector capacity limitations and plasmid incompatibility. For example, the pyrimidine biosynthetic operon from *B. subtilis* was introduced into the *yghX* locus of *E. coli* with the help of two exogenous protospacer and protospacer-adjacent motifs and two corresponding gRNA plasmids [11]. We divided the *Bspur* operon into four parts and integrated them into different neutral sites using the CRISPR/Cas9-mediated homologous recombination method. The introduced *Bspur* operon was used to enhance the enzymatic activities of purine nucleotide synthesis pathway. However, we could not detect the accumulation of guanosine in MQ4 (Fig. 2). In addition, the reaction of R5P to PRPP is considered a rate-limiting step for purine and pyrimidine nucleoside synthesis. *prs* overexpression also did not lead to detectable guanosine accumulation. Unexpectedly, the titre of inosine was significantly increased by overexpression of the *pur* operon and *prs*, indicating that high purine metabolic flux is directed towards inosine synthesis in cells (Fig. 3). Subsequently, the accumulation of guanosine was observed by disrupting degradation-related genes (∆*deoD*, ∆*ppnP* and ∆*gsk*), and MQ19 obtained 41.5 mg/L guanosine (Fig. 4). These results indicated the necessity of blocking product catabolism pathways for the target products, which was in accord with previous reports [11, 35]. Notably, because guanosine synthesis involves multiple precursors, enhancing the supply of other precursors, such as glutamine and aspartate, should be taken into consideration in the future.

Metabolic flux redistribution is an effective strategy to enhance chemical synthesis. In this study, downregulating the flux of adenosine synthesis and the EMP and ED pathways drove more metabolic flux into guanosine biosynthesis, which illustrated a synergistic effect on guanosine production (Figs. 5 and 6). Optimization of central carbon metabolism can enhance the availability of precursors, thereby improving the titre, productivity and yield of the biosynthetic target compounds and enhancing the performance of engineered strains. This strategy has been used to enhance the titre of other chemicals, including nucleosides, terpenoids and fatty acid derivatives, amino acids, organic acids and natural products [15, 36–40]. For example, the titre of inosine was significantly improved by overexpressing the key enzyme Zwf and blocking two essential backflow nodes including the purine synthesis pathway towards the PPP, and from the PPP to the glycolysis [15]. A previous study illustrated that deleting the *pfkA*, *edd* and *eda* genes can result in an approximately 11-fold increase in riboflavin titre in shake flasks [41]. The *fbp* gene overexpression also led to a shift in metabolic flux from the glycolysis to the PPP and an increase in riboflavin titre [26]. Alternatively, we can fine-tune metabolic pathway through promoter replacement with different strengths, inducible promoters and quorum sensing system to dynamically regulate key metabolic nodes to increase guanosine production. Further engineering should be focused on balancing biomass and products by controlling the carbon flux of glucose into the glycolysis.

## Conclusions

In summary, guanosine production in *E. coli* engineered strains was progressively increased by combinatorial metabolic engineering strategies, which included overexpression of the *Bspur* operon and *prs* gene, blockage of the guanosine degradation pathway, downregulation of *purA* expression, elimination of feedback and transcription dual inhibition, redirection of the metabolic flux of the EMP and ED pathways, redox cofactor rebalancing and engineering of transporters and strengthening the guanosine synthesis. Ultimately, the final optimized strain MQ39 produced 289.8 mg/L guanosine. Combinatorial metabolic engineering strategies would be beneficial to further engineer *E. coli* to act as an excellent chassis strain for industrial guanosine production in the future.

### Electronic supplementary material

Below is the link to the electronic supplementary material.

Supplementary Material 1

## Data Availability

No datasets were generated or analysed during the current study.

## Abbreviations

ED: Entner-Doudoroff
EMP: Embden–Meyerhof–Parnas
PPP: pentose phosphate pathway
CRISPR/Cas9: Clustered regularly interspaced short palindromic repeats (CRISPR)/CRISPR-associated protein 9 (Cas9)
PRPP: 5′-phosphoribosyl pyrophosphate
R5P: ribose-5′-phosphate
PCR: polymerase chain reaction
HPLC: High-performance liquid chromatography
IPTG: isopropyl-β-thiogalactopyranoside
DNS: 3,5-dinitrosalicylic acid
G6P: Glucose-6-phosphate
F6P: fructose-6-phosphate
6-PG: 6-phosphogluconate
FBP: fructose-1,6-diphosphate
ADSS: adenylosuccinate synthetase
