# Supplementary material for: Efficient production of guanosine in Escherichia coli by combinatorial metabolic engineering
Source: Microb Cell Fact. 2024 Jun 19;23:182. doi: 10.1186/s12934-024-02452-8 (PMC11186194; doi:10.1186/s12934-024-02452-8)

**Supplementary Information**

**Efficient production of** **guanosine in *Escherichia coli* by combinatorial metabolic engineering**

Kun Zhang, Mengxing Qin, Yu Hou, Wenwen Zhang, Zhenyu Wang, Hailei Wang*

Henan Province Engineering Laboratory for Bioconversion Technology of Functional Microbes, College of Life Sciences, Henan Normal University, Xinxiang 453007, China

* Correspondence author. E-mail addresses: whl@htu.cn

**Table S1. Primers used in this study**

| **Primers** | **Sequence（5ʹ-3΄）** |
| --- | --- |
| *pSC101*-P*_BAD_*-T-F | GCACAGATGCGTAAGGAGA |
| *pSC101*-P*_BAD_*-T-R | CGCGGAACCCCTATTTGTTT |
| *pSC101*-P*_BAD_*-T-TF | GCGACACGGAAATGTTGAAT |
| *pSC101*-P*_BAD_*-T-TR | CGCGTACCATGGGATCCTTA |
| *lacI*-sgRNA | GAGACATCAAGAAATAACGCCGG |
| *lacI*sg-F | GAGACATCAAGAAATAACGCGTTTTAGAGCTAGAAATAGCAAGTT |
| *lacI*sg-R | CTAAAACGCGTTATTTCTTGATGTCTCATGGAGAAACAGTAGAGAGTTG |
| *lacI* UP-F | AAAAATAAACAAATAGGGGTTCCGCGCCGGCGTTATTTCTTGATGTCTCAGATAACTGCCGTCACTCCA |
| *lacI* UP-R | GCATGATAGCGCCCGGAAGAGAGTCAATTCTCACTCATTAGGCACCCCAG |
| *lacI* DN-F | GAATTGACTCTCTTCCGGGC |
| *lacI* DN-R | GGTATTTTCTCCTTACGCATCTGTGCCCGGCGTTATTTCTTGATGTCTCCGTGACGAACAGTGGATTTC |
| *lacI* UP-F1 | GCTCATCCGCCACATATCCT |
| *lacI* DN-R1 | GACTGACCATCAAAGTGCGG |
| *yghX*-sgRNA | CTGGACACCCGAATCAACGAGGG |
| *yghX*sg-F | CTGGACACCCGAATCAACGAGTTTTAGAGCTAGAAATAGCAAGTT |
| *yghX*sg-R | CTAAAACTCGTTGATTCGGGTGTCCAGATGGAGAAACAGTAGAGAGTTG |
| *yghX* UP-F | AAAAATAAACAAATAGGGGTTCCGCGCCCTCGTTGATTCGGGTGTCCAGATCGATGCGCCATTTACCAC |
| *yghX* UP-R | CCTCCTCATTATACGAGCCGGATGATTAATTGTCAACCCGTTATGACAAATCTGCCG |
| *purE*-F | TTGACAATTAATCATCCGGCTCGTATAATGAGGAGGATGCAGCCGCTAGTAGGAATC |
| *purK*-R | AAACCCCGCCCTGTCAGGGGCGGGGTTTTTTTTTTCATTCTGCTTGTCCTCCGTCT |
| *yghX* DN-F | AAAAAAAAACCCCGCCCCTGACAGGGCGGGGTTTTTTTTGCCACCGCCATAGCAAAATC |
| *yghX* DN-R | GGTATTTTCTCCTTACGCATCTGTGCCCCTCGTTGATTCGGGTGTCCAGAGAGTGACGAAAAACTGGGC |
| *yghX* UP-F1 | AACCGATTCGGCCATCTCTG |
| *yghX* DN-R1 | GCCATAAACCGAGCGATAACG |
| *ypjC*-*ileY*-sgRNA | GCCATTTCAAGGAAGAAGTGCGG |
| *ypjC*-*ileY*sg-F | GCCATTTCAAGGAAGAAGTGGTTTTAGAGCTAGAAATAGCAAGTT |
| *ypjC*-*ileY*sg-R | CTAAAACCACTTCTTCCTTGAAATGGCATGGAGAAACAGTAGAGAGTTG |
| *ypjC* UP-F1 | TCCACTACCCAAAACTGCGC |
| *ileY* DN-R1 | CAATGTGGGGTTGTCGATAGG |
| *ypjC* UP-F | AAAAATAAACAAATAGGGGTTCCGCGCCGCACTTCTTCCTTGAAATGGCAACTGCTTCACTTGCTACGC |
| *ypjC* UP-R | CCTCCTCATTATACGAGCCGGATGATTAATTGTCAAGGAAAAAATGCCTGCTATGCC |
| *purB*-F | GACAATTAATCATCCGGCTCGTATAATGAGGAGGATGATCGAACGTTATTCAAGACCTG |
| *purQ*-R | CGAAAAAACCCCGCCGAAGCGGGGTTTTTTGCGTCAAGCAGTAGTGACATGAGTTTCC |
| *ileY* DN-F | AAAAAACCCCGCTTCGGCGGGGTTTTTTCGCAATGATTTGGTGGCCCTTGC |
| *ileY* DN-R | GGTATTTTCTCCTTACGCATCTGTGCCCGCACTTCTTCCTTGAAATGGCTCGATGTGGTCTGGAGAGGC |
| *fliK*-sgRNA | TGCCATTCGTGAGAACCCAGCGG |
| *fliK*sg-F | TGCCATTCGTGAGAACCCAGGTTTTAGAGCTAGAAATAGCAAGTT |
| *fliK*sg-R | CTAAAACCTGGGTTCTCACGAATGGCAATGGAGAAACAGTAGAGAGTTG |
| *fliK* UP-F1 | CGGCACTGCTTGCAGAAAAC |
| *fliK* DN-R1 | GACCGTGAAGGTATCCAGCG |
| *fliK* UP-F | AAAAATAAACAAATAGGGGTTCCGCGCCGCTGGGTTCTCACGAATGGCACAGGCGAGACAACTACCGAC |
| *fliK* UP-R | CCTCCTCATTATACGAGCCGGATGATTAATTGTCAAGGTGTGCTGATGACTTCCGC |
| *purL*-F | TTGACAATTAATCATCCGGCTCGTATAATGAGGAGGATGTCACTACTGCTTGAACCAAG |
| *purF*-R | CCGAAGCGGGGTTTTCGGCGGAACTGCGCTGCCTTTATGTC |
| *fliK* DN-F | AGTTCCGCCGAAAACCCCGCTTCGGCGGGGTTTTGCCGCCCCGCAGGATTTAGGTGAAG |
| *fliK* DN-R | GGTATTTTCTCCTTACGCATCTGTGCCCGCTGGGTTCTCACGAATGGCACGAAAATATCAACGCCGCTG |
| *lacZ*-sgRNA | TCGCACAGCGTGTACCACAGCGG |
| *lacZ*sg-F | TCGCACAGCGTGTACCACAGGTTTTAGAGCTAGAAATAGCAAGTT |
| *lacZ*sg-R | CTAAAACCTGTGGTACACGCTGTGCGAATGGAGAAACAGTAGAGAGTTG |
| *lacZ* UP-F1 | GTCGCGTTCGGTTGCACTAC |
| *lacZ* DN-R1 | ACTCGGCGTTTCATCTGTGG |
| *lacZ* UP-F | AAAAATAAACAAATAGGGGTTCCGCGCCGCTGTGGTACACGCTGTGCGAGCTGCTGGTGTTTTGCTTCC |
| *lacZ* UP-R | CCTCCTCATTATACGAGCCGGATGATTAATTGTCAAGTGATCATCTGGTCGCTGGG |
| *purM*-F | ACAATTAATCATCCGGCTCGTATAATGAGGAGGATGTCTGAAGCATATAAAAACGCAGG |
| *purD*-R | CTCAAAAGGCCATCCGTCAGGATGGCCTTCTTTATTTTTGGGCAGCCTTTAAAGCGC |
| *lacZ* DN-F | AGAAGGCCATCCTGACGGATGGCCTTTTGAGGATGATGCTCGTGACGG |
| *lacZ* DN-R | GGTATTTTCTCCTTACGCATCTGTGCCCGCTGTGGTACACGCTGTGCGAAGGATATGTGGCGGATGAGC |
| *aslA*-*glmZ*-sgRNA | TCTGGCGCAGTTGATATGTAAGG |
| *aslA*-*glmZ*sg-F | TCTGGCGCAGTTGATATGTAGTTTTAGAGCTAGAAATAGCAAGTT |
| *aslA*-*glmZ*sg-R | CTAAAACTACATATCAACTGCGCCAGAATGGAGAAACAGTAGAGAGTTG |
| *aslA* UP-F1 | ATCAAAGCCAACGTTCTGCG |
| *glmZ* DN-R1 | CACGGAGAATGGCAGGAAGC |
| *aslA* UP-F | AAAAATAAACAAATAGGGGTTCCGCGTCTGGCGCAGTTGATATGTAAGGTCAGAATGCCGTGGTGGATG |
| *aslA* UP-R | CCTCCTCATTATACGAGCCGGATGATTAATTGTCAAGTAATCATCCTGTCAGGGAGAGG |
| *prs*-F | GACAATTAATCATCCGGCTCGTATAATGAGGAGGGTGCCTGATATGAAGCTTTTTGCTG |
| *prs*-R | CGAAACCCCGCCGAAGCGGGGTTTGCGGCGTTAGTGTTCGAACATGGCAGAGATC |
| *glmZ* DN-F | CAAACCCCGCTTCGGCGGGGTTTCGCCGCAAGTATGTGTAGGATCAAGCTCAGG |
| *glmZ* DN-R | GGTATTTTCTCCTTACGCATCTGTGCTCTGGCGCAGTTGATATGTAAGGTGGACAGACTGCACAAGCCG |
| *Ecprs^D128A^*-F | GACCGTGTGCTGACAGTGGCTCTGCACGCTGAACAGATTCAG |
| *Ecprs^D128A^*-R | TCTGTTCAGCGTGCAGAGCCACTGTCAGCACACGGTCAAC |
| *Ecprs^D128A^* UP-F | CTTTCCCCATTGCACAGAGC |
| *Ecprs^D128A^* DN-R | CGACGGTTATCCCGGCAATC |
| *Baprs*-F | ACAATTAATCATCCGGCTCGTATAATGAGGAGGATGTCTAACGAATACGGAGATAAG |
| *Baprs*-R | CGAAACCCCGCCGAAGCGGGGTTTGCGGCGTTAGCTGAATAAATAGCTGAC |
| *deoD*-sgRNA | GACCTGTTCTACTCTCCGGACGG |
| *deoD*sg-F | GACCTGTTCTACTCTCCGGAGTTTTAGAGCTAGAAATAGCAAGTT |
| *deoD*sg-R | CTAAAACTCCGGAGAGTAGAACAGGTCATGGAGAAACAGTAGAGAGTTG |
| *deoD* UP-F1 | CCAACGGCGGCTACAATATC |
| *deoD* DN-R1 | GGTAAATTTTGGCTGCTCGC |
| *deoD* UP-F | AAAAATAAACAAATAGGGGTTCCGCGCCGTCCGGAGAGTAGAACAGGTCAAGTGAAAGCGACTGGCCTG |
| *deoD* UP-R | ACAAGGCAATCGCCTTGCAGCGAAACACAATGTTTTATCCTTTTTGTGACATAACAAAG |
| *deoD* DN-F | TTGTGTTTCGCTGCAAGGCG |
| *deoD* DN-R | GGTATTTTCTCCTTACGCATCTGTGCCCGTCCGGAGAGTAGAACAGGTCCACTGTTCGTCGCCATCTGC |
| *rihA*-sgRNA | AAACAGGGTTACCAATCGCGCGG |
| *rihA*sg-F | AAACAGGGTTACCAATCGCGGTTTTAGAGCTAGAAATAGCAAGTT |
| *rihA*sg-R | CTAAAACCGCGATTGGTAACCCTGTTTATGGAGAAACAGTAGAGAGTTG |
| *rihA* UP-F1 | ACCGAGATCAAACACCAGCG |
| *rihA* DN-R1 | AAACCGATCTGGCAAAGCTG |
| *rihA* UP-F | AAAAATAAACAAATAGGGGTTCCGCGCCGCGCGATTGGTAACCCTGTTTAGCGTACCGCATTTAACCCG |
| *rihA* UP-R | CACACGTCATTGATTAAGATCACCACG |
| *rihA* DN-F | ACGCGTGGTGATCTTAATCAATGACGTGTGTGTTGCTCCTTGTTGTGTGCTTC |
| *rihA* DN-R | GGTATTTTCTCCTTACGCATCTGTGCCCGCGCGATTGGTAACCCTGTTTCCTGAAACTGCTGGAGCGTG |
| *rihB*-sgRNA | CATCACTAATGGAACGCCGGAGG |
| *rihB*sg-F | CATCACTAATGGAACGCCGGGTTTTAGAGCTAGAAATAGCAAGTT |
| *rihB*sg-R | CTAAAACCCGGCGTTCCATTAGTGATGATGGAGAAACAGTAGAGAGTTG |
| *rihB* UP-F1 | AAAGGGTTTGACGATTGCCG |
| *rihB* DN-R1 | GTGAATGAACTGCGCGAGAAC |
| *rihB* UP-F | AAAAATAAACAAATAGGGGTTCCGCGCCTCCGGCGTTCCATTAGTGATGTTCTGGAAGATACCGCCGAG |
| *rihB* UP-R | TAACGCAACTGGAAACAGAGGAAATAAACAGTGTTGATAACAAGCCGGGCC |
| *rihB* DN-F | TGTTTATTTCCTCTGTTTCCAGTTGCG |
| *rihB* DN-R | GGTATTTTCTCCTTACGCATCTGTGCCCTCCGGCGTTCCATTAGTGATGGCCAGGCGATTCACTAACGG |
| *rihC*-sgRNA | AGAAACGCCGGTATCCCGAGCGG |
| *rihC*sg-F | AGAAACGCCGGTATCCCGAGGTTTTAGAGCTAGAAATAGCAAGTT |
| *rihC*sg-R | CTAAAACCTCGGGATACCGGCGTTTCTATGGAGAAACAGTAGAGAGTTG |
| *rihC* UP-F1 | AAGTGGAAGGGACAATGGGC |
| *rihC* DN-R1 | TCACCCATCACTTTGGCTGC |
| *rihC* UP-F | AAAAATAAACAAATAGGGGTTCCGCGCCGCTCGGGATACCGGCGTTTCTGTCTGATGTGATCGACGCGC |
| *rihC* UP-R | GTTTTCTCCATAAAAAATGCCGGTAACAAG |
| *rihC* DN-F | CTTGTTACCGGCATTTTTTATGGAGAAAACCCTGTCACATGTTATTGGCATGCAG |
| *rihC* DN-R | GGTATTTTCTCCTTACGCATCTGTGCCCGCTCGGGATACCGGCGTTTCTTCGTCAAACCCCGTAGTGCC |
| *ppnP*-sgRNA | TTCAACCATAACACCCACGCTGG |
| *ppnP*sg-F | TTCAACCATAACACCCACGCGTTTTAGAGCTAGAAATAGCAAGTT |
| *ppnP*sg-R | CTAAAACGCGTGGGTGTTATGGTTGAAATGGAGAAACAGTAGAGAGTTG |
| *ppnP* UP-F1 | CGGCAAAGTGAAATCAATCGGC |
| *ppnP* DN-R1 | TTAGGTGGCGAAGCACAACG |
| *ppnP* UP-F | AAAAATAAACAAATAGGGGTTCCGCGCCAGCGTGGGTGTTATGGTTGAAGCAAAGTGGAGCGTGACCTG |
| *ppnP* UP-R | AAACTGGCCCTGTTTGGTAGTTG |
| *ppnP* DN-F | AGATGTTCAACTACCAAACAGGGCCAGTTTTTCCTCGCCTTCCCCTTGAAC |
| *ppnP* DN-R | GGTATTTTCTCCTTACGCATCTGTGCCCAGCGTGGGTGTTATGGTTGAAACTGCGAATTTTCCGCGTTG |
| *gsk*-sgRNA | TCATTGCCGAGAATCCGCAGTGG |
| *gsk*sg-F | TCATTGCCGAGAATCCGCAGGTTTTAGAGCTAGAAATAGCAAGTT |
| *gsk*sg-R | CTAAAACCTGCGGATTCTCGGCAATGAATGGAGAAACAGTAGAGAGTTG |
| *gsk* UP-F1 | AATCACCGGCAAAGCCAATG |
| *gsk* DN-R1 | GGCCGGACAAAACCTGGTAC |
| *gsk* UP-F | AAAAATAAACAAATAGGGGTTCCGCGCCACTGCGGATTCTCGGCAATGAGCGCTTCAGGTGAAAGGGTG |
| *gsk* UP-R | GGTTGTTTTTACGGGTGGTTAACTG |
| *gsk* DN-F | CCGCTCAGTTAACCACCCGTAAAAACAACCGTTATCGTCGGTTCGTAGGCC |
| *gsk* DN-R | GGTATTTTCTCCTTACGCATCTGTGCCCACTGCGGATTCTCGGCAATGATTACGGTCGTGTAGGCAGCC |
| *purA*-*sgRNA* | ACGCTGCTGGATATCGACCACGG |
| *purA*sg-F | ACGCTGCTGGATATCGACCAGTTTTAGAGCTAGAAATAGCAAGTT |
| *purA*sg-R | CTAAAACTGGTCGATATCCAGCAGCGTATGGAGAAACAGTAGAGAGTTG |

| *purA* UP-F1 | GCGGAAGTGCTGTTGAAACG |
| --- | --- |
| *purA* DN-R1 | CTCGGTTCCTCCGTTGTCATC |
| *purA* UP-F | AAAAATAAACAAATAGGGGTTCCGCGCCGTGGTCGATATCCAGCAGCGTTAACGGCTGAGACGAAGGGC |
| *purA* UP-R | TTTTCAAAATCACCGTTTGCTTAAAAATGGATTC |
| *purA* DN-F | AATCCATTTTTAAGCAAACGGTGATTTTGAAAATTCTGGTACGCCTGGCAGATATTTTG |
| *purA* DN-R | GGTATTTTCTCCTTACGCATCTGTGCCCGTGGTCGATATCCAGCAGCGTTTCAACCAAATCGGCAAGCG |
| *BspurA* UP-R | CATCCTCCTCATTATACGAGCCGGATGATTAATTGTCAACATTGGGTGCCCAGTACGAC |
| *BspurA*-F | AATTAATCATCCGGCTCGTATAATGAGGAGGATGTCTTCAGTAGTTGTAGTAGGTACGC |
| *BspurA*-R1 | TGACACCGCCGGCAACGTTGTTAGATGACGTAACAAACGGGTATGTTC |
| *BspurA*-F1 | TTTGTTACGTCATCTAACAACGTTGCCGGCGGTGTCAC |
| *BspurA*-R | GCGGAAAAACCCCGCCGAAGCGGGGTTTTTGGCGTTAGTTCGCACGGTACACACTGC |
| *BspurA* DN-F | AAAAACCCCGCTTCGGCGGGGTTTTTCCGCTTCTGGTACGCCTGGCAGATATTTTG |
| *BspurA* DN-R1 | CCCGACTAGGGATGGGATTC |
| *yjet*-sgRNA | CGATGCTTTACCCGAAGGCATGG |
| *yjet*sg-F | CGATGCTTTACCCGAAGGCAGTTTTAGAGCTAGAAATAGCAAGTT |
| *yjet*sg-R | CTAAAACTGCCTTCGGGTAAAGCATCGATGGAGAAACAGTAGAGAGTTG |
| *yjet* UP-F | AAAAATAAACAAATAGGGGTTCCGCGCCATGCCTTCGGGTAAAGCATCGTAACGGCTGAGACGAAGGGC |
| *yjet* UP-R | ATGACCTCGATCAGTCTGTTTTTTGTTCATTATCCTTATAGAAAAAGAAAACCACCGAC |
| *purA*-*ssrA*-F | AAGGATAATGAACAAAAAACAGACTGATCGAGGTCATTTTTG |
| *purA*-*ssrA*-R | GGCGTCCGCGTAGTTCTCGGAGTAGTTCTCGTCGTTGGCGGCCGCGTCGAACGGGTCGC |
| *yjet* DN-F | GACGAGAACTACTCCGAGAACTACGCGGACGCCAGCTAATTCTGGTACGCCTGGCAGAT |
| *yjet* DN-R | GGTATTTTCTCCTTACGCATCTGTGCCCATGCCTTCGGGTAAAGCATCGTTCAACCAAATCGGCAAGCG |
| *purA*-*ssrA*-TF | CCGCCAACGACGAGAACTAC |
| *purR*-sgRNA | AAGACGGCAGTAGGGCGATGCGG |
| *purR*sg-F | AAGACGGCAGTAGGGCGATGGTTTTAGAGCTAGAAATAGCAAGTT |
| *purR*sg-R | CTAAAACCATCGCCCTACTGCCGTCTTATGGAGAAACAGTAGAGAGTTG |
| *purR* UP-F1 | CGATGGCGGAAAGTACGTTG |
| *purR* DN-R1 | GGCGCGGTCAGTTAGCTATC |
| *purR* UP-F | AAAAATAAACAAATAGGGGTTCCGCGCCGCATCGCCCTACTGCCGTCTTAACAGGAAGGAGATGCGAGG |
| *purR* UP-R | GTTCAATCAAGCGCGGATGCTCCATTTCACTCCAGACCCTAACTTC |
| *purR* DN-F | GTGAAATGGAGCATCCGCGCTTGATTGAAC |
| *purR* DN-R | GGTATTTTCTCCTTACGCATCTGTGCCCGCATCGCCCTACTGCCGTCTTTTGTTGCCAGTCACCACCCG |
| *pfkA*-sgRNA | TCTGACATGATCAACCGTGGCGG |
| *pfkA*sg-F | TCTGACATGATCAACCGTGGGTTTTAGAGCTAGAAATAGCAAGTT |
| *pfkA*sg-R | CTAAAACCCACGGTTGATCATGTCAGAATGGAGAAACAGTAGAGAGTTG |
| *pfkA* UP-F1 | TAAAGCTGAGTCCCTCGCGG |
| *pfkA* DN-R1 | CAATGGTGGAAGTGTACGGTGC |
| *pfkA* UP-F | AAAAATAAACAAATAGGGGTTCCGCGCCGCCACGGTTGATCATGTCAGACTGGGGTTGTCCTGGTACGG |
| *pfkA* UP-R | CTGCCTTTTTCCGAAATCAGACTACCTCTGAACTTTGGAATGCAAAATG |
| *pfkA* DN-F | CATTTTGCATTCCAAAGTTCAGAGGTAGTCTGATTTCGGAAAAAGGCAGATTCCTTTAC |
| *pfkA* DN-R | GGTATTTTCTCCTTACGCATCTGTGCCCGCCACGGTTGATCATGTCAGAACCGACGTCGCTTGTTTACC |
| *EcglpX* UP-R | TCCTGCTAGCATAGTCCCTAGGACTGAGCTAGCTGTCAACCGCTTGTCAACACACCGAT |
| *EcglpX*-F | CAGCTAGCTCAGTCCTAGGGACTATGCTAGCAGGAGGATGAGACGAGAACTTGCCATCG |
| *EcglpX*-R | GCGGAAAAACCCCGCCGAAGCGGGGTTTTTGGCGTCAGAGGATGTGCACCTGCATTTC |
| *glpX* DN-F | AAAAACCCCGCTTCGGCGGGGTTTTTCCGCTGATTTCGGAAAAAGGCAGATTCCTTTAC |
| *glpX*-TF | GCTCAGTCCTAGGGACTATGCTAG |
| *BsglpX*-F | GCTCAGTCCTAGGGACTATGCTAGCAGGAGGATGGAAAGAAGTTTATCAATGGAATTGG |
| *BsglpX*-R | GAAAAACCCCGCCGAAGCGGGGTTTTTGGCGTTATGGACGGATTACAAGATTTGGTTTC |
| *edad*-sgRNA | GCATGAAGATGTCAATACGGTGG |
| *edad* UP-F1 | TGCACCAGTAGGTCATCGCC |
| *edad* DN-R1 | ATGCCTATGAACGTTTGCTGC |
| *edad*sg-F | GCATGAAGATGTCAATACGGGTTTTAGAGCTAGAAATAGCAAGTT |
| *edad*sg-R | CTAAAACCCGTATTGACATCTTCATGCATGGAGAAACAGTAGAGAGTTG |
| *edad* UP-F | AAAAATAAACAAATAGGGGTTCCGCGCCACCGTATTGACATCTTCATGCATGATACCGGGATGGTGACG |
| *edad* UP-R | GCTCTGCTTATCTCGCCCGGTACCGTGACTACCTGGCGCTG |
| *edad* DN-F | AGGTAGTCACGGTACCGGGCGAGATAAGCAGAGC |
| *edad* DN-R | GGTATTTTCTCCTTACGCATCTGTGCCCACCGTATTGACATCTTCATGCATGCGCCGAAACCGTATCAG |
| *pntAB*-sgRNA | CACGTAATAACCGACAACGCAGG |
| *pntAB*sg-F | CACGTAATAACCGACAACGCGTTTTAGAGCTAGAAATAGCAAGTT |
| *pntAB*sg-R | CTAAAACGCGTTGTCGGTTATTACGTGATGGAGAAACAGTAGAGAGTTG |
| *pntAB* UP-F1 | GCTCGAAGTGCCACAATTGC |
| *pntAB* DN-R1 | GGTTTAACCGCTGCCGCTTG |
| *pntAB* UP-F | AAAAATAAACAAATAGGGGTTCCGCGCCTGCGTTGTCGGTTATTACGTGGTTCAGTCCTCGCGGCAATC |
| *pntAB* UP-R | CGTGTTGCAGCAACGCCAAAACCGAAGAGTCCGATTGCTGG |
| *pntAB* DN-F | CCAGCAATCGGACTCTTCGGTTTTGGCGTTGCTGCAACACG |
| *pntAB* DN-R | GGTATTTTCTCCTTACGCATCTGTGCCCTGCGTTGTCGGTTATTACGTGATCCTTCGCCTTGCGCAAAC |
| *sthA*-UP-R | ATCCTCCTCATTATACGAGCCGGATGATTAATTGTCAACCGAAGAGTCCGATTGCTGG |
| *sthA*-F | GACAATTAATCATCCGGCTCGTATAATGAGGAGGATGCCACATTCCTACGATTACGATG |
| *sthA*-R | CCCGCCCTGTCAGGGGCGGGGTTTGCGGCGTTAAAACAGGCGGTTTAAACCGTTTAACG |
| *sthA*-DN-F | CCGCAAACCCCGCCCCTGACAGGGCGGGGTTTCGCCGCTTTTGGCGTTGCTGCAACACG |
| *yghE*-sgRNA | ACCACTTATGGCGAACCGGAAGG |
| *yghE*sg-F | ACCACTTATGGCGAACCGGAGTTTTAGAGCTAGAAATAGCAAGTT |
| *yghE*sg-R | CTAAAACTCCGGTTCGCCATAAGTGGTATGGAGAAACAGTAGAGAGTTG |
| *yghE* UP-F1 | CTGATGACCCGCGATACCTG |
| *yghE* DN-R1 | GGAAATCAACCGCGACCATG |
| *yghE* UP-F | AAAAATAAACAAATAGGGGTTCCGCGCCTTCCGGTTCGCCATAAGTGGTCTGTTGCTCAATCCGTTCGC |
| *yghE* UP-R | ATCCTCCTCATTATACGAGCCGGATGATTAATTGTCAATTATGGAGCGTCAGCGAACAG |
| *yghE* DN-F | CGCAAACCCCGCCCCTGACAGGGCGGGGTTTCGCCGCGAACATGACCGGAGCCAGC |
| *yghE* DN-R | GGTATTTTCTCCTTACGCATCTGTGCCCTTCCGGTTCGCCATAAGTGGTGTCGGTTATGCAGCGAAGCC |
| *nupG*-sgRNA | GGTAAACAGAACCAGAATGGCGG |
| *nupG*sg-F | GGTAAACAGAACCAGAATGGGTTTTAGAGCTAGAAATAGCAAGTT |
| *nupG*sg-R | CTAAAACCCATTCTGGTTCTGTTTACCATGGAGAAACAGTAGAGAGTTG |
| *nupG* UP-F1 | TAACCCGTATGCGGTCAGCC |
| *nupG* DN-R1 | TGGTTGAGGTGTTGCCGAGC |
| *nupG* UP-F | AAAAATAAACAAATAGGGGTTCCGCGCCGCCATTCTGGTTCTGTTTACCAAGATGTGTTCCGCTCGCAG |
| *nupG* UP-R | CATACGGTCTGCCAGTCGGTGTTAATTTCCTCACATCGTGATGCGG |
| *nupG* DN-F | CCGCATCACGATGTGAGGAAATTAACACCGACTGGCAGACCGTATG |
| *nupG* DN-R | GGTATTTTCTCCTTACGCATCTGTGCCCGCCATTCTGGTTCTGTTTACCTGCGCCAGTTGTGTCAGGAG |
| *nupG* UP-R1 | TCCTGCTAGCATTATACCTAGGACTGAGCTAGCTGTCAGCAGCCAACTTCCCCACAGAC |
| *nepI*-F | TAGCTCAGTCCTAGGTATAATGCTAGCAGGAGGATGAGTGAATTTATTGCCGAAAACCG |
| *nepI*-R | AAAACCCCGCCGAAGCGGGGTTTTTGGCGTCAGGATTTCTTCATTTTCACCTTTGCAG |
| *nupG* DN-F1 | CCAAAAACCCCGCTTCGGCGGGGTTTTTCCGCACCGACTGGCAGACCGTATG |
| *nepI*-TF | TTATCCAGCGCATAACCGCC |
| *phuE*-F | TAGCTCAGTCCTAGGTATAATGCTAGCAGGAGGATGAATTTCAAAGTTTTCCTGCTTGC |
| *phuE*-R | AAAACCCCGCCGAAGCGGGGTTTTTGGCGTTACAAGGAGGATTTTTTTGCTGTTTGAAC |
| *phuE*-TF | GTGTTTGCGCTGGGATATGC |
| *phuE*-TR | CCAAAACAACACCGCCAATG |
| *ykgH-betA-*sgRNA | GAATCCTTATATAAACACTGAGG |
| *ykgH-betA*sg-F | GAATCCTTATATAAACACTGGTTTTAGAGCTAGAAATAGCAAGTT |
| *ykgH*-*betA*sg-R | CTAAAACCAGTGTTTATATAAGGATTCATGGAGAAACAGTAGAGAGTTG |
| *ykgH* UP-F1 | ATCCCAGAGCCAGGCAAATG |
| *ykgH* UP-F | AAAAATAAACAAATAGGGGTTCCGCGCCTCAGTGTTTATATAAGGATTCTCATCGAGAACTTGCCTGCC |
| *ykgH* UP-R | TCCTCCTCATTATACGAGCCGATGATTAATTGTCAAACCCGAACATTCCTGATACAGAC |
| *guaB*-F | TGACAATTAATCATCGGCTCGTATAATGAGGAGGATGCTACGTATCGCTAAAGAAGCTC |
| *guaA*-R | GAAACCCCGCCCTGTCAGGGGCGGGGTTTGCGGCGTCATTCCCACTCAATGGTAGCTGG |
| *betA* DN-F | CAAACCCCGCCCCTGACAGGGCGGGGTTTCGCCGCCAATCTGATCGGTTCCTGCG |
| *betA* DN-R | GGTATTTTCTCCTTACGCATCTGTGCCCTCAGTGTTTATATAAGGATTCCGGCTCGAATGCAGTGAAAG |
| *betA* DN-R1 | GTGAGGAATTTGCGTGGCCG |
| *guaC*-sgRNA | GAGTTGTACAAACAGAACCTGGG |
| *guaC*sg-F | GAGTTGTACAAACAGAACCTGTTTTAGAGCTAGAAATAGCAAGTT |
| *guaC*sg-R | CTAAAACAGGTTCTGTTTGTACAACTCATGGAGAAACAGTAGAGAGTTG |
| *guaC* UP-F1 | AGTTGCGTTTCTGGGCTGAC |
| *guaC* UP-F | AAAAATAAACAAATAGGGGTTCCGCGCCCAGGTTCTGTTTGTACAACTCTTGCTGGATCTGGTGTTGCG |
| *guaC* UP-R1 | CATCCTCCTCATTATACGAGCCGATGATTAATTGTCAAGTGCGGATTCCTGGGGTTAAT |
| *guaC* DN-F1 | CAAACCCCGCCCCTGACAGGGCGGGGTTTCGCCGCTCTCCCAACGCTGGCGTGGA |
| *guaC* DN-R | GGTATTTTCTCCTTACGCATCTGTGCCCCAGGTTCTGTTTGTACAACTCTTGTTCGGCTTTCTGCTGGC |
| *guaC* DN-R1 | AAAAAGCCCAGCGTCAGTTG |
| *guaC* UP-R | AGTTGCGTTTCTGGGCTGAC |
| *guaC* DN-F | TACCAGCAATGGCACAACCC |

**Fig.S1. Structure diagram of integration expression cassettes used in this study**

**
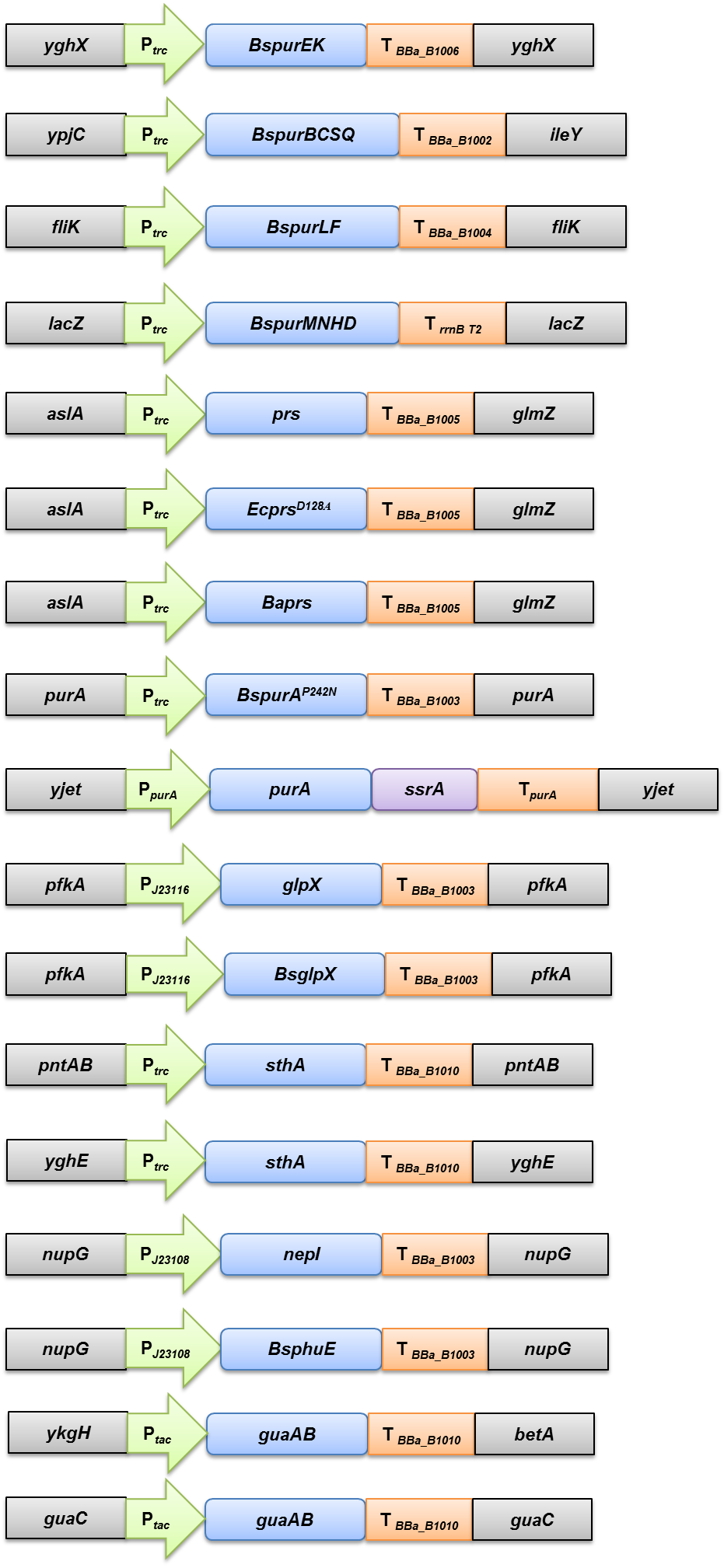
**

**Fig.S2. Schematic diagram of the genotype evolution of guanosine engineered strains**


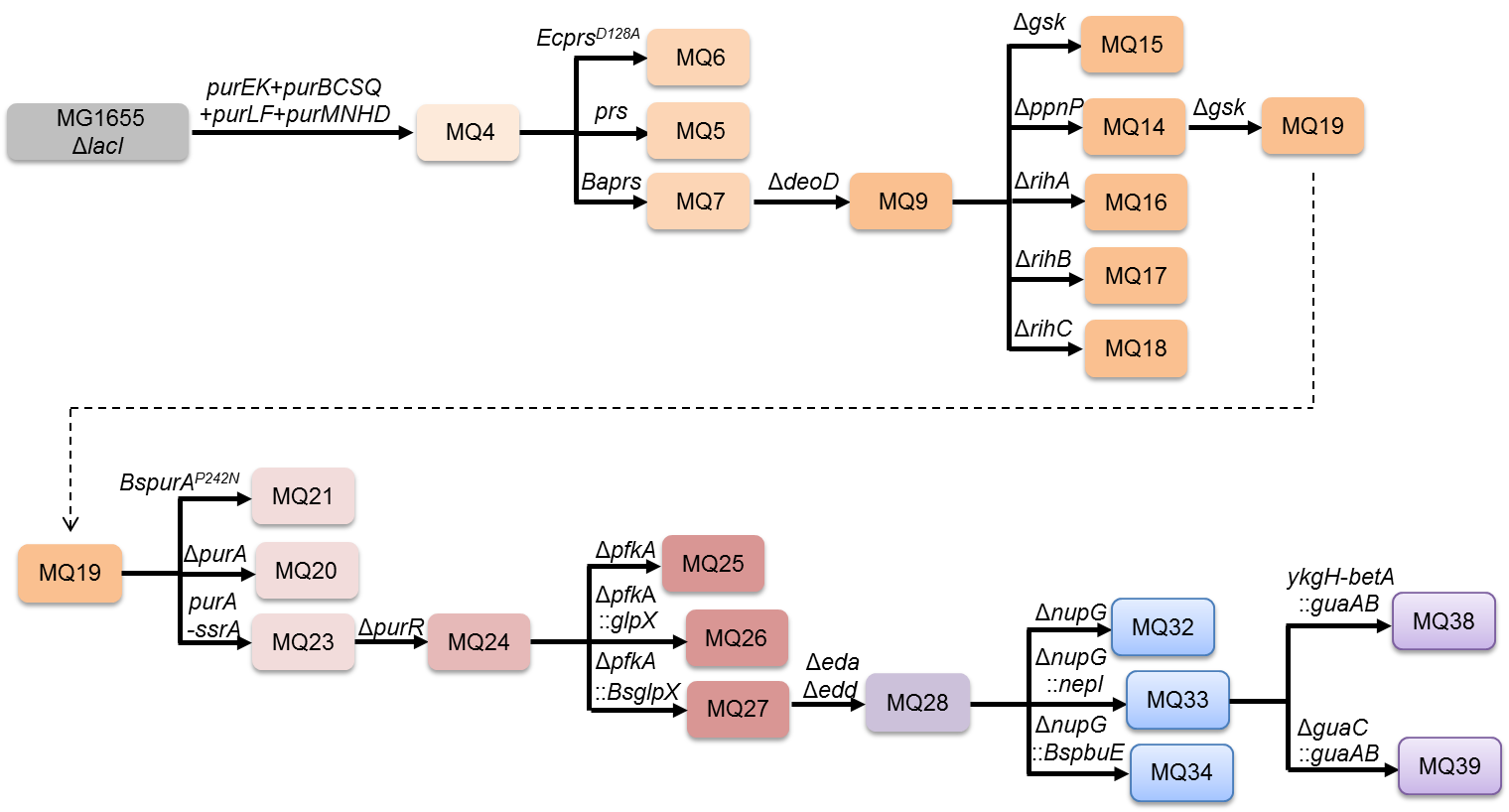

Supplement: Supplementary file 1 — Supplementary Material 1 [file 12934_2024_2452_MOESM1_ESM.docx]
